# Risk predictors of progression to severe disease during the febrile phase of dengue: a systematic review and meta-analysis

**DOI:** 10.1016/S1473-3099(20)30601-0

**Published:** 2021-07

**Authors:** Sorawat Sangkaew, Damien Ming, Adhiratha Boonyasiri, Kate Honeyford, Siripen Kalayanarooj, Sophie Yacoub, Ilaria Dorigatti, Alison Holmes

**Affiliations:** a Section of Adult Infectious Disease, Department of Infectious Disease, Faculty of Medicine, Imperial College London, London, UK; b Global Digital Health Unit, Department of Primary Care and Public Health, School of Public Health, Imperial College London, London, UK; c MRC Centre for Global Infectious Disease Analysis, Department of Infectious Disease Epidemiology, School of Public Health, Imperial College London, London, UK; d Department of Social Medicine, Hatyai Hospital, Songkhla, Thailand; e Department of Pediatrics, Queen Sirikit National Institute of Child Health, Bangkok, Thailand; f Oxford University Clinical Research Unit, Wellcome Trust Major Overseas Programme, Ho Chi Minh City, Vietnam; g Centre for Tropical Medicine and Global Health, University of Oxford, Oxford, UK; h Antimicrobial Resistance Collaborative, Imperial College London, London, UK

## Abstract

**Background:**

The ability to accurately predict early progression of dengue to severe disease is crucial for patient triage and clinical management. Previous systematic reviews and meta-analyses have found significant heterogeneity in predictors of severe disease due to large variation in these factors during the time course of the illness. We aimed to identify factors associated with progression to severe dengue disease that are detectable specifically in the febrile phase.

**Methods:**

We did a systematic review and meta-analysis to identify predictors identifiable during the febrile phase associated with progression to severe disease defined according to WHO criteria. Eight medical databases were searched for studies published from Jan 1, 1997, to Jan 31, 2020. Original clinical studies in English assessing the association of factors detected during the febrile phase with progression to severe dengue were selected and assessed by three reviewers, with discrepancies resolved by consensus. Meta-analyses were done using random-effects models to estimate pooled effect sizes. Only predictors reported in at least four studies were included in the meta-analyses. Heterogeneity was assessed using the Cochrane Q and *I*^2^ statistics, and publication bias was assessed by Egger's test. We did subgroup analyses of studies with children and adults. The study is registered with PROSPERO, CRD42018093363.

**Findings:**

Of 6643 studies identified, 150 articles were included in the systematic review, and 122 articles comprising 25 potential predictors were included in the meta-analyses. Female patients had a higher risk of severe dengue than male patients in the main analysis (2674 [16·2%] of 16 481 *vs* 3052 [10·5%] of 29 142; odds ratio [OR] 1·13 [95% CI 1·01–1·26) but not in the subgroup analysis of studies with children. Pre-existing comorbidities associated with severe disease were diabetes (135 [31·3%] of 431 with *vs* 868 [16·0%] of 5421 without; crude OR 4·38 [2·58–7·43]), hypertension (240 [35·0%] of 685 *vs* 763 [20·6%] of 3695; 2·19 [1·36–3·53]), renal disease (44 [45·8%] of 96 *vs* 271 [16·0%] of 1690; 4·67 [2·21–9·88]), and cardiovascular disease (nine [23·1%] of 39 *vs* 155 [8·6%] of 1793; 2·79 [1·04–7·50]). Clinical features during the febrile phase associated with progression to severe disease were vomiting (329 [13·5%] of 2432 with *vs* 258 [6·8%] of 3797 without; 2·25 [1·87–2·71]), abdominal pain and tenderness (321 [17·7%] of 1814 *vs* 435 [8·1%] of 5357; 1·92 [1·35–2·74]), spontaneous or mucosal bleeding (147 [17·9%] of 822 *vs* 676 [10·8%] of 6235; 1·57 [1·13–2·19]), and the presence of clinical fluid accumulation (40 [42·1%] of 95 *vs* 212 [14·9%] of 1425; 4·61 [2·29–9·26]). During the first 4 days of illness, platelet count was lower (standardised mean difference −0·34 [95% CI −0·54 to −0·15]), serum albumin was lower (−0·5 [–0·86 to −0·15]), and aminotransferase concentrations were higher (aspartate aminotransferase [AST] 1·06 [0·54 to 1·57] and alanine aminotransferase [ALT] 0·73 [0·36 to 1·09]) among individuals who progressed to severe disease. Dengue virus serotype 2 was associated with severe disease in children. Secondary infections (*vs* primary infections) were also associated with severe disease (1682 [11·8%] of 14 252 with *vs* 507 [5·2%] of 9660 without; OR 2·26 [95% CI 1·65–3·09]). Although the included studies had a moderate to high risk of bias in terms of study confounding, the risk of bias was low to moderate in other domains. Heterogeneity of the pooled results varied from low to high on different factors.

**Interpretation:**

This analysis supports monitoring of the warning signs described in the 2009 WHO guidelines on dengue. In addition, testing for infecting serotype and monitoring platelet count and serum albumin, AST, and ALT concentrations during the febrile phase of illness could improve the early prediction of severe dengue.

**Funding:**

Wellcome Trust, National Institute for Health Research, Collaborative Project to Increase Production of Rural Doctors, and Royal Thai Government.

## Introduction

Dengue poses a large burden on public health systems worldwide. In the estimated burden of dengue in 2010, 70% of the global incidence of dengue infection occurred in south and southeast Asia, followed by Latin America and the western Pacific region.^1^ In 2009 to 2013, dengue epidemics were reported in some parts of subtropical regions, such as in Europe, the USA, and China.^2^, ^3^ The global incidence has been estimated at 390 million infected individuals each year.^1^ Of those, an estimated 96 million individuals have symptomatic infections and 10 000–20 000 individuals die from dengue annually.^1^, ^4^ Although the majority of infected individuals are asymptomatic or experience a benign febrile illness, a minority develop a life-threatening syndrome, known as severe dengue or dengue haemorrhagic fever. The progression to severe disease commonly occurs after the febrile phase, between days 4 and 6 of illness.^5^ Early detection of disease progression during the febrile phase, therefore, has a major role in improving case management and reducing the health-care burden of dengue.^5^

**Research in context**

**Evidence before this study**

We searched MEDLINE and PROSPERO for meta-analyses, systematic reviews, and reviews up to Jan 31, 2018. The search terms were “dengue”, and one of: “dengue haemorrhage fever”, “severe dengue”, or “severity”. Only reports written in English were included. Previous systematic reviews and meta-analyses of predictors for progression to severe disease have considered the whole time course of illness and have not differentiated the febrile phase (typically days 1–4 of illness) from the later clinical phases when severe manifestations occur. Because of variation in clinical parameters during the time course of the illness, these previous studies might have missed significant associations between early prognostic factors and progression to severe disease. In addition, not all the warning signs in the 2009 WHO dengue guideline are likely to be of use in the early prediction of severe dengue, because factors such as clinical fluid accumulation or increasing haematocrit occur late relative to the critical phases. The aim of this study was to identify factors associated with progression to severe dengue disease that are detectable specifically in the febrile phase.

**Added value of this study**

In line with the 2009 WHO guidelines, we found that vomiting, abdominal pain and tenderness, spontaneous and mucosal bleeding, and clinical fluid accumulation were clinical features associated with severe disease. In addition, we found that the presence of specific pre-existing comorbidities (diabetes, hypertension, and renal disease) were associated with progression to severe disease. During the first 4 days of febrile illness, individuals with a lower platelet count and serum albumin, and with higher aminotransferase concentrations (aspartate aminotransferase and alanine aminotransferase) were more likely to progress to severe disease. The infection of dengue serotype 2 among children and secondary infections were also associated with progression to severe disease.

**Implications of all the available evidence**

Our findings support the use of warning signs described in the 2009 WHO guideline. They also indicate that the signs are dynamic, varying during the phases of disease, and that risk assessment in the early febrile phase of disease could be enhanced with the inclusion of the additional prognostic signs we have identified. Improving the prediction of progression to severe disease could considerably improve the management of patients and health-care resource allocation in endemic areas.

To assist clinicians in the early detection of severe disease progression, a Special Programme for Research and Training in Tropical Diseases, in collaboration with WHO, recommended the use of warning signs detailed in the 2009 WHO dengue guideline^5^ as early indicators of plasma leakage. These warning signs are abdominal pain or tenderness, persistent vomiting, clinical fluid accumulation, mucosal bleeding, lethargy or restlessness, liver enlargement by more than 2 cm, and an increase in haematocrit concurrent with rapid decrease in platelet count. Although the use of these warning signs achieves high sensitivity in detecting those at risk of disease progression during the febrile phase, it substantially increases the number of unnecessary admissions, particularly in endemic areas and during epidemics.^6^, ^7^ In addition, some of the warning signs occur late relative to onset of plasma leakage, and therefore have limited clinical value.

Although hundreds of clinical studies have been done to identify factors associated with the early onset of plasma leakage, most have included a small number of patients, and overall their results remain inconclusive. To address this issue, systematic reviews and meta-analyses have been done.^8^, ^9^, ^10^ These studies have found high heterogeneity in the clinical factors associated with disease progression, most likely due to considerable variation in the clinical time course considered in the studies. As a result, early clinical factors (ie, those occurring during the febrile phase) associated with disease progression have yet to be defined.

In this systematic review and meta-analysis, we aimed to identify early predictors in the disease time course that are associated with progression to severe dengue disease. The results of this analysis could provide insight into early prognostic factors associated with severe disease progression and support the optimisation of patient triage to improve quality of care.

## Methods

### Search strategy and selection criteria

For this systematic review and meta-analysis, we followed the protocol described in the PRISMA-P guidelines^11^ and the steps discussed in the guide to systematic review and meta-analysis of prognostic factor studies by Riley and colleagues.^12^

We searched six medical databases (MEDLINE, Embase, Global Health, CINAHL, Web of Science, and Scopus) for publications on predictors of severe dengue or dengue haemorrhagic fever from Jan 1, 1997, to Jan 31, 2020. For relevant grey literature we searched Open Grey, and for relevant dissertations we searched ProQuest Dissertations and Theses Global. The search terms and full eligibility criteria are described in the appendix (pp 2–3). The search was restricted to original articles written in English (books, conference abstracts, comments, notes, and letters were excluded) and was adjusted for each database. Medical subject headings terms and the explode feature were used when applicable (appendix p 1). The search terms and strategy were verified by a medical reference librarian to ensure that the syntax was correct and complete.

Duplicate articles were removed using EndNote X8 software (Clarivate Analytics, Philadelphia, PA, USA), and the list of remaining articles was subsequently checked for duplicates manually. Abstract screening and full-text selection were done independently by two reviewers (SS and DM) using predefined eligibility criteria (appendix p 2) and Covidence software (Veritas Health Innovation, Melbourne, VIC, Australia). Discrepancies between the two reviewers were resolved by a third reviewer (AB), and the final decision was then made by consensus. To be included in the meta-analysis, studies had to report associations between predictors detected during the febrile phase of dengue or the first 4 days of illness and dengue severity. In addition, predictors included in the meta-analysis had to be reported in at least four studies.

### Data extraction and quality assessment

Data extraction was done using a standardised data extraction form (appendix p 4). For factors reported in at least four studies, we extracted crude measures of association from univariable analyses and adjusted measures of association from multivariable models. Articles that reported the same hospital of recruitment, period of study, and associated factors were identified as duplicates, in which case only the study with the largest sample size was included.

To assess the quality and risk of bias of the studies, the Quality in Prognostic Factor Studies (QUIPS) tool was used.^13^ Two reviewers (SS and either DM or AB) assessed the quality independently. Discrepancies were resolved by discussion. Data extraction and study quality assessment were done using Microsoft Excel 365.

### Data analysis

We did a meta-analysis to combine the effect sizes of factors potentially associated with disease progression using R version 3.4.3 and the R package meta.^14^, ^15^ Combining the classifications of severe illness from the 1997 and 2009 WHO guidelines,^5^, ^16^ we divided dengue progression into two groups: individuals who progressed to severe disease (defined as dengue haemorrhagic fever according to 1997 guidelines or severe dengue according to 2009 guidelines) and individuals who did not progress to severe disease (dengue fever and non-severe dengue; appendix pp 5–7). Each risk factor was analysed separately in univariable meta-analysis. For continuous variables, we standardised the sample means and SDs.^17^, ^18^ Effect sizes and SEs were calculated using Hedge's g, which is a bias-corrected standardised mean difference. The effect sizes from the univariable analyses and multivariable models were pooled separately. Random-effects models were used to generate pooled estimates because populations were all assumed to be different. The DerSimonian–Laird method was used to estimate the between-study variance. In addition, we did subgroup analyses of studies in children (study population age <18 years) and adults (≥18 years) to identify prognostic factors specific to those populations. The age cutoff in some studies was inconsistent and not always explicitly defined. For example, patients aged between 14 years and 18 years were classified as adults in some studies and as children in others. We assigned studies as being in adults or children based on the definition used within each study.

Heterogeneity between studies was assessed using the Cochran Q statistic and the *I*^2^ statistic. Studies were considered to be heterogeneous at a p value for the Cochran Q statistic of less than 0·1; the levels of heterogeneity were categorised as low (*I*^2^<25%), low to moderate (25% to <50%), moderate to high (50% to <75%), or high (≥75%).^19^, ^20^ If heterogeneity was suspected, subgroup analysis was done if there were more than four studies in each subgroup. The heterogeneity of the pooled effect sizes among subgroups was also considered with the Cochran Q statistic. The agreement between reviewers in study selection was assessed using Cohen's κ.

To investigate the robustness of the pooled estimates, a sensitivity analysis was done in which studies with extreme effect sizes and heterogeneity were removed. A sensitivity analysis on the effect of platelet count was done by removing the two studies reporting odds ratios (ORs) instead of standardised mean differences (SMDs). Finally, the small-study effect was assessed to detect any publication bias using visual inspection of funnel plots and Egger's test. Publication bias was suspected when the p value of the Egger's test was less than 0·05.^21^ We used Duval and Tweedie's trim and fill method^22^ to assess the sensitivity of the crude estimates to publication bias and to adjust the pooled effect sizes accordingly.

We did a dose-response meta-analysis for age using the R package dosresmeta.^23^ Both linear and non-linear models were fitted to estimate trends in the risk of progression to severe disease with age. The Akaike information criterion and the Bayesian information criterion were used to select the optimal model.

The study was registered with PROSPERO, CRD42018093363.

### Role of the funding source

The funders of the study had no role in study design, data collection, data analysis, data interpretation, or writing of the report. All authors had full access to all the data in the study and had final responsibility for the decision to submit for publication.

## Results

**Figure 1:**
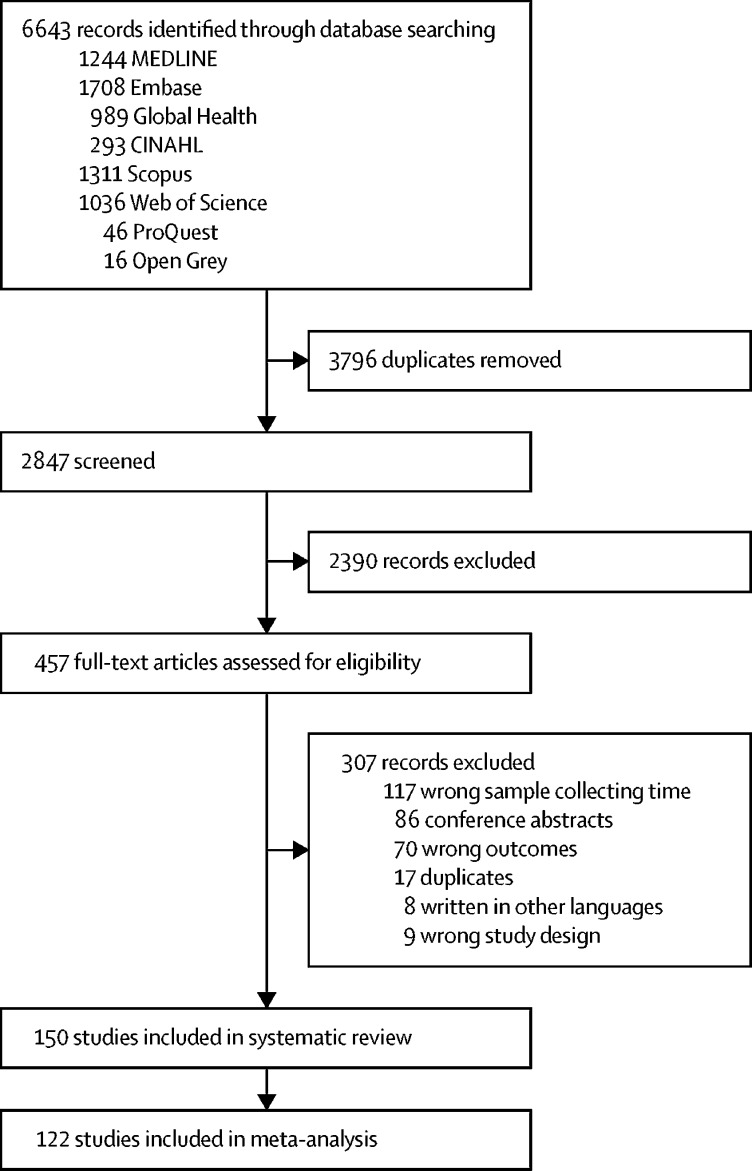
Flow diagram of study selection

Our initial searches identified 6643 articles, of which 150 were included in the systematic review and 122 were included in the meta-analysis (figure 1). In the selection of studies, agreement between the two reviewers was moderate (κ index 0·70 [95% CI 0·63–0·77] and 87% agreement [95% CI 82–90]). More than 80% of the included studies (121 of 150) were done in Asia and Latin America and published between 2008 and 2020. 60% (90 of 150) of the selected studies were done using a cohort study design, 29% (44 of 150) were nested case-control, case-cohort, or case-control studies, and the remaining 11% (16 of 150) were cross-sectional studies. Around 60% (87 of 150) of the studies defined severity outcomes using the 1997 WHO classification,^16^ and the remaining studies used the 2009 WHO classification.^5^ Details of the studies included in the systematic review and meta-analysis are presented in the appendix (p 8).

Among the 150 studies included in the systematic review, we identified 203 factors tested for their association with disease progression, 25 of which were reported in at least four studies and were thus included in the meta-analyses. These factors were grouped into four domains: demographics and comorbidities (nine factors), clinical signs and symptoms (seven factors), patient laboratory parameters (seven factors), and viral-related biomarkers (two factors). The definitions of identified factors included in this meta-analysis are in the appendix (p 34–37). Here, we report the pooled crude associations for all 25 factors, and the pooled adjusted associations for age, diabetes, and vomiting. A summary of our findings is presented in the table.

**Table:** Summary of factors detectable within the first 4 days of illness and their association with progression to severe dengue disease

	**Associated with progression**	**Not associated with progression**
Demographics and comorbidity	Older age (in adults), younger age (in children), female sex, diabetes, hypertension, renal disease, cardiovascular disease	Nutritional status, bodyweight, mixed comorbidity
Signs and symptoms	Vomiting, abdominal pain and tenderness, bleeding, and pleural effusion or ascites	Rash, headache, and positive tourniquet test
Laboratory parameters	Platelet count, aspartate aminotransferase, alanine aminotransferase, serum albumin, and secondary infection	White blood cell count, haematocrit
Virological profile	Dengue viral serotype 2 (in children)	Viral load

**Figure 2:**
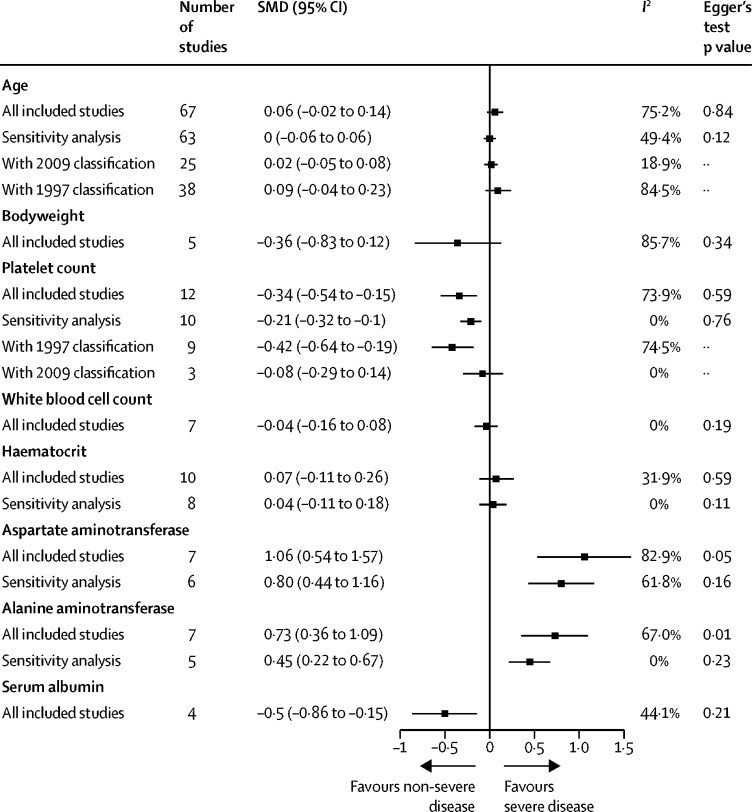
Pooled SMD of factors potentially associated with progression to severe dengue In sensitivity analyses of all factors except platelet count, studies were excluded due to extreme effect sizes and heterogeneity. For platelet count, the sensitivity analysis was done excluding studies that reported odds ratios instead of SMDs. SMD=standardised mean difference.

**Figure 3:**
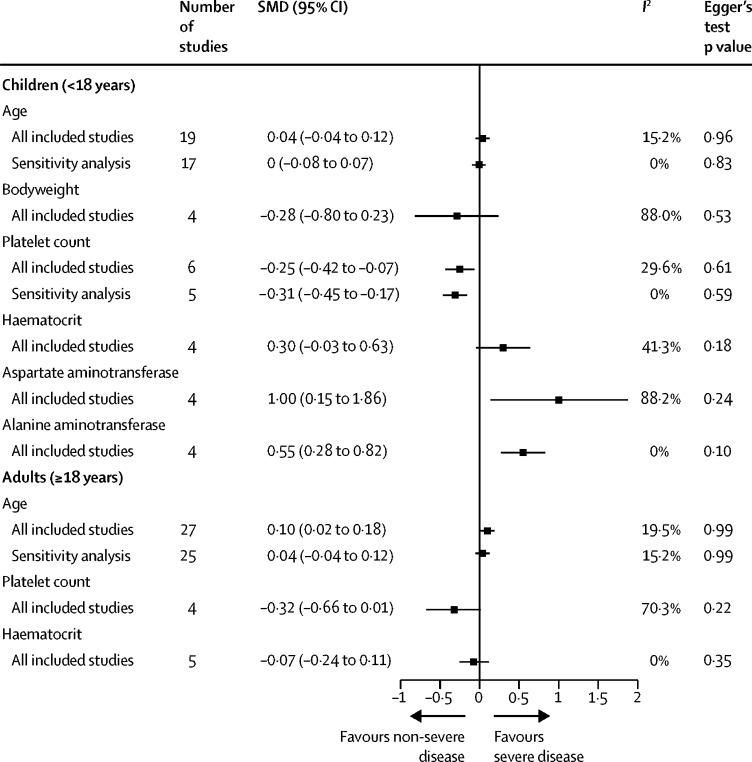
Pooled SMD of factors potentially associated with progression to severe dengue by age of study population In sensitivity analyses, studies were excluded due to extreme effect sizes and heterogeneity. SMD=standardised mean difference.

The age profile of patients who progressed to severe disease was not significantly different from the age profile of patients who did not (figure 2). Heterogeneity in the pooled results was high in the main meta-analysis and low to moderate in the sensitivity analysis that excluded four studies with extreme effect sizes and heterogeneity (figure 2). In the subgroup analysis of studies by age group, adults who progressed to severe disease were significantly older than adults who did not (SMD 0·1 [95% CI 0·02–0·18]; figure 3). For the adjusted associations of age from multivariable models, all included studies were done in children and used the 1997 WHO classification. We found that younger children were at higher risk of developing dengue haemorrhagic fever than older children, with the odds of developing dengue haemorrhagic fever reducing by 8% (95% CI 2–14) for every year of increase in age (appendix p 40). In a dose-response meta-analysis, we found that a linear model between the OR of disease progression and age achieved the lowest Akaike information criterion and Bayesian information criterion, with an OR of 1·03 (95% CI 0·96–1·11) for each year of increase in age, but this result was not statistically significant (appendix pp 41–42).

**Figure 4:**
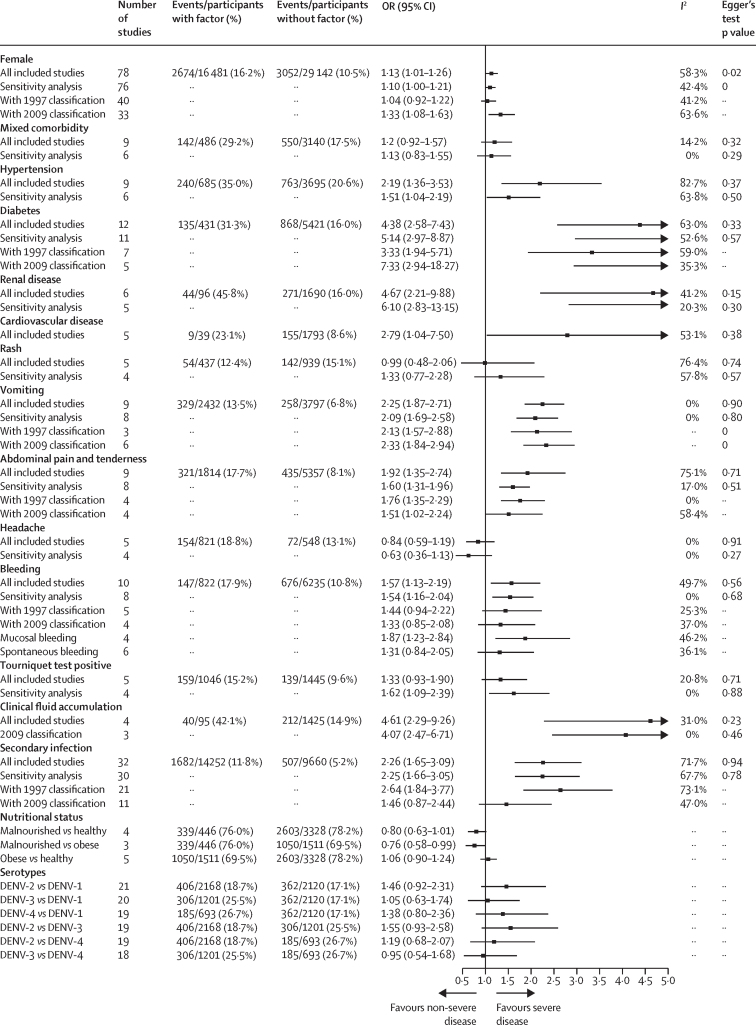
Pooled ORs of progression to severe dengue given the presence of potential prognostic factors In sensitivity analyses, studies were excluded due to extreme effect sizes and heterogeneity. DENV=dengue virus serotype. OR=odds ratio.

**Figure 5:**
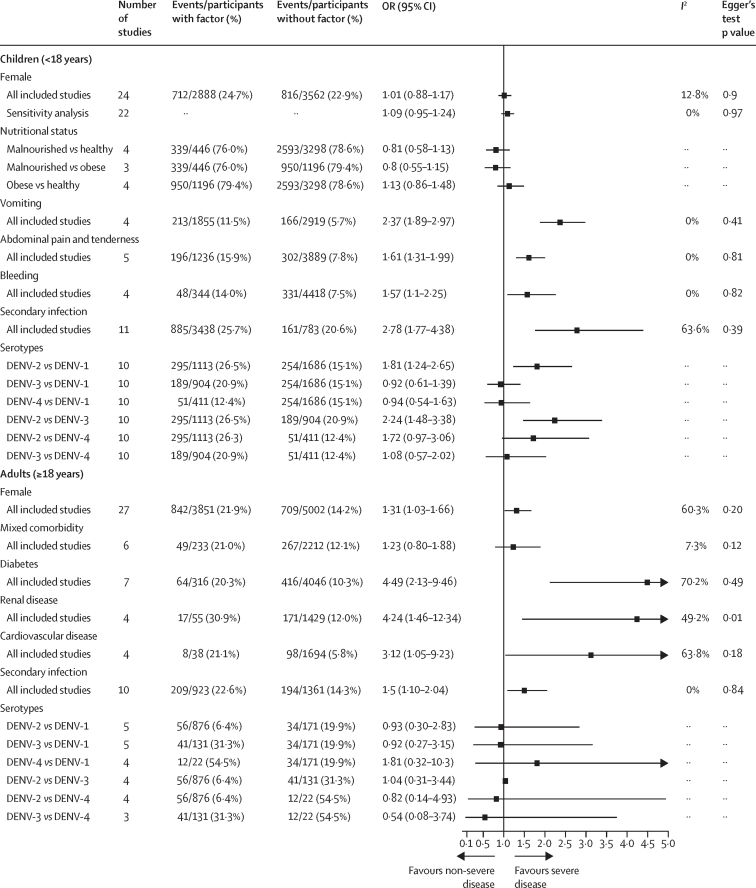
Pooled OR of progression to severe dengue given the presence of potential prognostic factors by age of study population In sensitivity analyses, studies were excluded due to extreme effect sizes and heterogeneity. DENV=dengue virus serotype. OR=odds ratio.

The association between sex and progression to severe disease was significant in the main analysis but not in a sensitivity analysis in which two studies were removed due to their extreme effect sizes and heterogeneity (figure 4).^24^, ^25^ There was evidence of publication bias in the studies assessing sex as a predictor of disease progression that were included in the meta-analysis (Egger's test p=0·021). The pooled OR after use of the trim and fill method to account for publication bias was 0·95 (95% CI 0·84–1·08; appendix p 43). In the subgroup analysis by age, female sex was associated with a higher risk of progression to severe disease in adults but not in children (figure 5).

The presence of mixed comorbidity was not associated with progression to severe disease (figure 4). Hypertension, diabetes, renal disease, and cardiovascular disease were positively associated with progression to severe dengue (figure 4), with associations strongest for diabetes (OR 4·38 [95% CI 2·58–7·43]) and renal disease (4·67 [2·21–9·88]). For the pooled ORs of the four comorbidities, heterogeneity was moderate to high or high in both the main analyses and sensitivity analyses, with the exception of renal disease, for which heterogeneity was low to moderate in the main analysis and low in the sensitivity analysis. As with the pooled adjusted OR taken from multivariable models, the presence of diabetes also increased the risk of progression to severe disease, with an OR of 1·87 (1·30–2·73).

Five studies reported associations between nutritional status and progression to severe disease, four of which included only children. For children, nutritional status was based on their bodyweight for age, according to local standardised guidelines, whereas for adults, body-mass index (BMI) was used (malnourished defined as a BMI of <18 kg/m^2^ and obese defined as a BMI of >30 kg/m^2^). We found some evidence that malnourishment was associated with a reduced risk of developing severe dengue and obesity was associated with an increased risk of progression to severe disease, although neither association was significant (figure 4). In comparison with individuals with malnourishment, individuals with obesity were at a significantly higher risk of developing severe disease.

In terms of clinical signs and symptoms, we found that presenting with vomiting, abdominal pain and tenderness, bleeding, or clinical fluid accumulation was associated with an increased risk of progression to severe disease (figure 4). The definition of vomiting was not given in most of the included studies, although two of eight studies defined vomiting as at least two episodes during the febrile phase of the illness.^25^, ^26^ For vomiting, a significant association was found in both pooled analyses (crude and adjusted estimates; figure 4; appendix p 43), and neither heterogeneity nor publication bias was found. Abdominal pain and tenderness were also consistently associated with progression to severe disease (OR 1·92 [95% CI 1·35–2·74]), although the definition was not given in all included studies and high heterogeneity was found (*I*^2^≥75%).

Individuals presenting with bleeding (defined as mucosal or spontaneous) had a higher risk of progressing to severe disease than did individuals without bleeding (figure 4). We did a sensitivity analysis to evaluate the potential effect of the two bleeding definitions; the difference between the pooled ORs obtained using the two definitions (mucosal or spontaneous) was not significant (Q statistic p=0·26). The positive association between bleeding and progression to severe disease was also consistent in the sensitivity analysis (ie, having removed two studies^27^, ^28^), with no heterogeneity observed in the sensitivity analysis.

For clinical fluid accumulation (either pleural effusion or ascites detectable during the first 4 days of illness), four studies were included; three referred to both ascites and pleural effusion^25^, ^26^, ^29^ and one referred to pleural effusion only.^30^ Despite the small number of studies included in this analysis and little information on the method used for detecting effusion, we found a significant association between clinical fluid accumulation during the first 4 days of illness and progression to severe dengue, with low to moderate heterogeneity (*I*^2^=31%; figure 4).

Rash and headache were not significantly associated with progression to severe disease, with no evidence of publication bias. Heterogeneity was not found in the pooled results of headache but was high in the pooled results of rash. A positive tourniquet test was not significantly associated with progression to severe disease and we found low heterogeneity and no publication bias in the pooled results of this sign. In the sensitivity analysis, a positive tourniquet test was associated with progression to severe disease, with an OR of 1·62 (95% CI 1·09–2·39).

The haematological parameters of haematocrit, white blood cell count, and platelet count were included in the meta-analyses. Of these, only platelet count was significantly associated with progression to severe disease when measured during the febrile phase (figure 2). Of the 12 studies assessing platelet count as a predictor of disease progression that were included in the meta-analysis, two reported ORs^26^, ^31^ which were transformed and pooled with the SMDs reported in the other studies. The pooled SMD of platelet count was significantly lower among individuals who progressed to severe disease than among individuals who did not. This finding was supported by the result of a sensitivity analysis in which we omitted the two studies originally reporting ORs (figure 2).^32^, ^33^ We also found a significant association between platelet count and progression to severe disease in a subgroup analysis of studies that used the 1997 WHO classification (figure 2) and a subgroup analysis of studies in children (figure 3). We found neither heterogeneity nor publication bias in the sensitivity analysis. We found no significant association between haematocrit levels or white blood cell count and progression to severe disease in any of the analyses (Figure 2, Figure 3).

Seven studies were included in the meta-analysis of aminotransferase concentrations. One study reported ORs for associations between disease progression and aspartate aminotransferase (AST) or alanine aminotransferase (ALT) abnormality, with a normal cutoff value at 40 units per dL.^30^ The ORs were first transformed into SMDs and then pooled with the other effect sizes.^34^ Meta-analyses assessing the associations of disease progression with AST or ALT showed that higher concentrations of these enzymes during the febrile phase were associated with progression to severe disease (figure 2). These positive associations were supported by the results of sensitivity analyses in which we omitted the studies reporting the results with the greatest effect sizes (one study for AST^35^ and two studies for ALT;^30^, ^36^ figure 2). We found publication bias in both meta-analyses for AST and ALT (appendix p 44–45). However, the adjusted pooled SMDs after use of the trim and fill method were significant (AST, 0·81 [95% CI 0·20–1·42]; ALT, 0·73 [0·36–1·09]).

Four studies were included in the meta-analysis of serum albumin, all of which used the 1997 WHO classification (no studies using the 2009 WHO definition reported associations with serum albumin). All studies consistently reported that individuals who progressed to dengue haemorrhagic fever had lower serum albumin concentrations than individuals who did not progress to dengue haemorrhagic fever during the febrile phase, with low to moderate heterogeneity and no publication bias (figure 2).

Secondary infection with dengue virus (versus primary infection) was significantly associated with progression to severe dengue (figure 4), particularly in children (figure 5). The significant association remained in a sensitivity analysis in which we omitted the two studies with the largest effect sizes and most heterogenous associations.^37^, ^38^ The heterogeneity in the pooled results of the main analysis and sensitivity analysis were moderate to high. In the subgroup analysis by age, heterogeneity was moderate to high in adults and no heterogeneity was found in children.

Four studies were included in the meta-analysis of viraemia, which found no significant association between viraemia and progression to severe disease (appendix p 46).^35^, ^39^, ^40^, ^41^ We found high heterogeneity in these studies (*I*^2^=95%; appendix p 46). Two of the four studies found no significant association between viraemia and progression to severe disease, one study showed that higher viraemia was associated with severe disease, and one study found that lower viraemia was associated with severe disease. In one study, a positive association between viraemia and progression to severe dengue was observed in adults with secondary infections with dengue virus serotypes 1, 2, and 3 (DENV-1, DENV-2, and DENV-3), whereas a negative association was found in children with secondary infections with DENV-1.^35^

In the meta-analysis of viral serotypes, DENV-2 tended to be associated with progression to severe dengue compared with other serotypes in the main analysis, but the findings were not significant (figure 4). DENV-2 was significantly associated with progression to severe disease in the subgroup analysis of studies in children (figure 5). Children infected with DENV-2 were at a higher risk of developing severe dengue than children infected with DENV-1 (OR 1·81 [95% CI 1·24–2·65]) or DENV-3 (2·24 [1·48–3·38]; figure 5).

Using the QUIPS tool to assess risk of bias in six domains (appendix p 47), we found a high risk of bias in terms of potential confounders not being addressed and adjusted for appropriately. There was also a considerable risk of bias in terms of patient participation because some studies recruited patients from the inpatient department, which could have missed some patients presenting with mild symptoms. The risk of bias in terms of study attrition was low. Although the risk of bias in terms of measurement of outcomes and prognostic factors was low because the included studies used definitions based on WHO guidelines, 25% of included studies were considered to have a moderate risk of bias for outcomes and prognostic factors.

## Discussion

In this systematic review and meta-analysis, we identified that younger age in children, older age in adults, and female sex were demographical risk factors for progression to severe disease. Pre-existing diabetes, hypertension, renal disease, cardiovascular disease, and presenting with vomiting, abdominal pain and tenderness, bleeding, or clinical fluid accumulation during the febrile phase of illness, were also associated with progression to severe disease. In addition, DENV-2 infection among children, secondary infection, a lower platelet count, lower serum albumin, and higher AST and ALT concentrations detected during the febrile phase were significantly associated with progression to severe disease (table).

The finding of a higher risk of progression to severe disease in older adults could be due to co-existing diabetes, hypertension, or renal disease, which are common in older adults. In the meta-analysis of studies in children, a pooled OR from multivariable models, which were already adjusted for immune response, showed that younger children were at a higher risk of progression to severe disease, which might be due to increased vascular filtration capacity among younger children.^42^

Female sex was associated with progression to severe disease in the main analysis and in a subgroup analysis of studies in adults. The higher risk of progression to severe dengue in women could be in part due to different health-seeking behaviours or differences in the immune response between men and women.^43^, ^44^

Diabetes and hypertension were included as risk factors for progression to severe illness in the 1997 and 2009 WHO guidelines.^5^, ^16^ The findings from our meta-analysis support inclusion of these risk factors. Although the pathophysiology underlying the contribution of diabetes to progression of dengue is not fully understood, factors including pre-existing vasculature damage and associated endothelial activation might contribute. Lee and colleagues^45^ found that patients with poor glycaemic control (HbA_1c_ >7%), with or without an additional comorbidity, were at a higher risk of developing dengue haemorrhagic fever and dengue shock syndrome than were patients with diabetes with adequate glycaemic control and no additional comorbidity. Our meta-analysis also showed positive associations between underlying renal disease or cardiovascular disease and progression to severe dengue. Chronic kidney disease could result in increased concentrations of pro-inflammatory cytokines, which could add to the risk of vascular injury during dengue infections.^46^ Uraemia, commonly found in chronic kidney disease, also causes endothelial dysfunction, predisposing individuals to worse dengue-associated vasculopathy.^47^, ^48^ However, associations between renal disease or cardiovascular disease and disease progression should be interpreted with caution because they were not adjusted for potential confounders, such as diabetes or hypertension, which are major causes of chronic kidney disease and cardiovascular disease.

Several clinical signs or symptoms were associated with progression to severe disease. We found a robust association with vomiting, with no heterogeneity, whereas high heterogeneity was found in the meta-analysis of abdominal pain and tenderness. This finding is probably due to the subjective nature of the symptom, which depends on both the patient and the physician doing the clinical examination. Bleeding as a clinical entity varies widely from gum bleeding to haematochezia or melaena. However, the presence of minor bleeding (ie, mucosal and spontaneous bleeding) during the febrile phase could be a prognostic sign for progression to severe disease.

All patients with dengue develop a spectrum of plasma leakage, and the presence of either pleural effusion or ascites, which can be classified as clinical fluid accumulation, can be a consequence of severe plasma leakage in the critical phase. However, the clinical detection of pleural effusion or ascites can reflect an increased risk of decompensation in the critical phase. Clinical fluid accumulation is particularly useful when the effusion is detected early within the febrile phase, before severe dengue develops. It will also increase in utility with time, given that the use of ultrasonography in low-income and middle-income countries is increasing and thresholds of detection of pleural effusion or ascites will decrease due to increased sensitivity of detection by ultrasound.

Although the use of a tourniquet test is recommended for diagnosing dengue haemorrhagic fever in the 1997 and 2009 WHO guidelines,^5^, ^16^ we found no evidence that this test allows early prediction of progression to severe disease. All studies of tourniquet test and progression used the 1997 WHO classification, in which the positive tourniquet test itself is a diagnostic criterion for dengue haemorrhagic fever. The positive association obtained in the sensitive analysis could therefore reflect this bias.

Haematological analyses have been used for dengue diagnosis and severity classification in a variety of health-care settings, from primary to tertiary. Platelet count was significantly associated with disease progression in our study, with patients with low platelet count showing a higher risk of progressing to severe disease. This finding is consistent with the observed decrease in platelet counts recorded in other observational studies^49^ and confirms platelet count as one of the key WHO warning signs.^5^ Although this meta-analysis could not provide robust clinical evidence of a platelet count cutoff to determine the risk of severe dengue, the majority of the average platelet counts in non-severe dengue groups were higher than 100 000 cells per dL. This value is in line with recommendations from the 1997 WHO dengue guideline and 2011 WHO South-East Asia region (SEARO) dengue guideline.^50^

Our analysis shows that higher concentrations of AST and ALT in the febrile phase are significantly associated with severe dengue and supports their monitoring during the febrile phase. However, potential confounders should be taken into consideration, such as co-infection with chronic hepatitis viruses, which increases the likelihood of hepatic dysfunctions and is common in dengue-endemic countries. Regarding the cutoff values for AST and ALT concentrations, we suggest that concentrations higher than three times the upper limit of normal are associated with progression to severe disease on the basis of the majority of the average AST and ALT concentrations reported in the included studies.

In our meta-analysis, serum albumin concentration during the febrile phase was significantly lower in individuals with severe dengue than in those with uncomplicated dengue. This finding is consistent with vascular leakage, with early extravasation of albumin along with plasma. However, serum albumin concentration can vary depending on pre-existing nutritional status, and therefore its use as a clinical cutoff might be challenging in practice. We would support the 2011 SEARO dengue guideline recommendation that a serum albumin concentration of 3·5 g/dL or lower, or a reduction by 0·5 g/dL during the febrile phase compared with baseline, is associated with progression to severe disease.^50^

Haematocrit and white blood cell count within 4 days of disease onset were not associated with disease severity in the critical phase. However, monitoring haematocrit remains essential for detecting plasma leakage in the critical phase.

DENV-2 was significantly associated with progression to severe dengue among studies in children, but there was no significant association among studies in adults or in the main meta-analysis. Because crude pooled associations were presented, the association between DENV-2 and severity among studies in adults could be confounded by other factors (eg, immune status, the sequence of infecting serotypes, time interval between infections, and pre-existing comorbidities).

The association between secondary infection and development of severe dengue is well documented and most likely represents antibody-dependent enhancement, whereby non-neutralising antibodies from the primary infection increase viral uptake into cells and a higher host viral burden. The observed association between secondary dengue infection and severe dengue is in line with several previous studies,^51^ including a modelling study^52^ showing that 18% (95% CI 16–20) of primary infections and 41% (0·36–0·45) of secondary infections are severe. In addition, the association between secondary infection and severe dengue was notably weaker in the subgroup analysis among adults, compared with that in children. This finding supports the effect of age on secondary infection and disease severity^53^ and the hypothesis that tertiary and quaternary infections are generally milder than secondary infections.^54^, ^55^ However, methods to distinguish tertiary and quaternary infections from secondary dengue infection are not widely available outside of research settings and prospective research studies, although such methods would be useful clinically.^54^, ^56^ Immune status testing at one early timepoint (during days 1–4 of illness) is also challenging due to the difficulty in distinguishing primary from secondary infections without acute and convalescent samples. Secondary infections are associated with a higher risk of progression to severe disease, but until more robust point-of-care diagnostics are developed,^57^, ^58^ incorporating immune status into early prediction models is difficult. Such rapid diagnostic tests would also inform vaccination campaigns.^59^

We found that individuals with malnourishment tended to be less likely to progress to severe dengue, although the association was significant only when compared with individuals with obesity. People who are malnourished might have a suppressed cellular immune response, causing reduced organ and tissue injury.^60^ Although we found no significant association between obesity or bodyweight and progression to severe dengue, a systematic review published in 2013 found a significant risk of dengue shock syndrome in children with obesity.^10^ This inconsistency could be due to the different outcomes used (ie, dengue shock syndrome instead of dengue haemorrhagic fever or severe dengue).

We found high heterogeneity in the pooled results of viraemia, which could be explained by the large individual-level variability, with measurements being taken at different times during infection and with different measurement methods across laboratories. Although peak viraemia could be a more consistent and robust metric to assess the association with severe dengue, it is often missed by the time patients present in hospital. In addition, quantification of blood viraemia might not reflect the total body viral load sequestered intracellularly.

Our systematic review and meta-analysis focused on the febrile phase, thus identifying factors that can predict progression to severe disease soon after the onset of symptoms. We found lower heterogeneity in pooled effect sizes compared with previous studies^8^, ^9^, ^10^ due to the shorter and more consistent time course of illness considered and lower variation in the clinical parameters.

This study has limitations. Few studies reported adjusted effect sizes, so we mostly pooled crude effect sizes, which could have been overestimated due to confounders. In the few instances in which meta-analyses were done with a small number of studies, pooled effect sizes should be interpreted with caution. Some factors were not clearly defined and could therefore present inconsistencies among studies. The quality of the final diagnosis and the method used to assign the final diagnosis, including evidence supporting the diagnoses, were not always clearly mentioned in the included studies. The presence of effusion, presence of bleeding, and platelet counts are also clinical outcomes of dengue haemorrhagic fever, which could affect the associations between these three prognostic factors and progression to severe disease. However, subgroup analyses by classification were done when data were available. Genetic factors were not included in this meta-analysis because these have not been routinely analysed along with clinical outcomes, and the study design and methodologies adopted for this area differ to those used to analyse clinical outcomes. Direct comparisons between clinical outcomes and genetic factors are therefore difficult. Limiting the selection of studies to those written in English might also have reduced the precision of the estimates. However, more than 80% of included studies were done in southeast Asia and Latin America. Therefore, the findings of this study are likely to be representative of practice in endemic areas. In addition, our meta-analysis did not capture all pre-existing comorbidities (eg, sickle cell disease or malaria infection^61^, ^62^), the use of non-invasive clinical monitoring (eg, ultrasonography, arterial pulse waveform, or side-stream darkfield imaging), or the role of novel biomarkers in dengue, which are of great research interest and represent a promising avenue to better understand dengue pathogenesis and identify new biomarkers.^63^, ^64^, ^65^, ^66^ Unfortunately, the number of studies involving these biomarkers is small to date, and substantial heterogeneity in study designs hindered our ability to define roles for every clinical factor or novel biomarker.

In this study, we have shown that monitoring serum albumin, AST, and ALT during the febrile phase of illness, in addition to the warning signs detailed in the 2009 WHO guidelines, could enhance the ability to predict the risk of a patient developing severe dengue. In addition, identifying the infecting viral serotype and the immunological status (eg, primary *vs* post-primary infections) during the febrile phase could further improve the accuracy of the risk predictions. Further prospective studies focusing on the febrile phase of illness will allow investigation of the role of novel biomarkers and non-invasive methods, such as ultrasonography, in predicting the early onset of severe dengue, with the ultimate objective of improving patient triage and allocation of resources, and reducing hospital morbidity, mortality, and the health and economic impact of dengue worldwide.

## Data sharing

Data will be made available upon request made to the corresponding author. The analysis code used in this study is available online.
