# Supplementary material for: Risk predictors of progression to severe disease during the febrile phase of dengue: a systematic review and meta-analysis
Source: Lancet Infect Dis. 2021 Jul;21(7):1014–26. doi: 10.1016/S1473-3099(20)30601-0 (PMC8240557; doi:10.1016/S1473-3099(20)30601-0)

THE LANCET
Infectious Diseases

Supplementary appendix

This appendix formed part of the original submission and has been peer reviewed. We post it as supplied by the authors.

Supplement to: Sangkaew S, Ming D, Boonyasiri A, et al. Risk predictors of progression to severe disease during the febrile phase of dengue: a systematic review and meta-analysis. *Lancet Infect Dis* 2021; published online Feb 25. https://doi.org/10.1016/S1473-3099(20)30601-0.

Supplement table 1: Search terms used in the literature review. We searched for all combinations of the terms in column 1 and 2 (e.g. "dengue shock syndrome AND risk factor(s)", "dengue shock syndrome AND risk parameter(s)", "dengue shock syndrome AND risk variable(s)", "dengue shock AND risk factor(s)" etc).

Complicated dengue	Predictors
- dengue shock syndrome- dengue shock<br>- dengue haemorrhagic fever/dengue<br>- hemorrhagic fever<br>- dengue with warning sign(s)<br>- severe dengue<br>- complicated dengue<br>- dengue severity	- risk factor(s)<br>- risk parameter(s)<br>- risk variable(s)<br>- predictive/predicting factor(s)<br>- predictive/predicting parameter(s)<br>- prognostic factor(s)<br>- prognostic parameter(s)<br>- Associated factor(s)<br>- Associated parameter(s)<br>- Associated characteristic(s)<br>- Risk(s)<br>- Prognosis<br>- Biomarker(s)<br>- Marker(s)

Supplement table 2: Search syntaxes applied in the Ovid platform for searching MEDLINE database. We searched for all combinations of the terms in column 1 and 2. The explode command (exp) was used to retrieve records that contained the specific term and its subheadings (e.g. Severe dengue, risk factors, and prognosis). Terms in the columns were search using multiple-purpose command (.mp), searching keywords in a set of fields usually including Title, Original Title, Abstract, and Subject Heading.

1. Terms for dengue with progression to severe disease	2. Terms for predictors
1. [exp Severe Dengue/]<br>2. severe dengue.mp.<br>3. dengue shock syndrome*.mp.<br>4. dengue h?emorrhagic fever.mp<br>5. dengue with warning sign*.mp.<br>6. dengue severity.mp.<br>7. dengue death.mp.<br>8. dengue death.mp.<br>9. dengue shock.mp.<br>10. 1 or 2 or 3 or 4 or 5 or 6 or 7 or 8 or 9	11. [exp Risk Factors/]<br>12. risk factor*.mp.<br>13. [exp RISK/]<br>14. risk*.mp.<br>15. [exp PROGNOSIS/]<br>16. prognosis.mp.<br>17. [exp BIOMARKERS/]<br>18. biomarker*.mp.<br>19. marker*.mp.<br>20. ((risk* or predictive or prognostic or associated) adj (factor* or parameter* or characteristic* or variable*)).mp. [mp=title, abstract, full text, caption text]<br>21. predictor*.mp.<br>22. prediction.mp.<br>23. 11 or 12 or 13 or 14 or 15 or 16 or 17 or 18 or 19 or 20 or 21 or 22
10 AND 23	

Supplement table 2: Template used to assess the eligibility criteria.

Reviewer name:	Date:
Author name/Study ID:	Year:
Title	Journal:
Study designs	**Include**<br>☐ Original papers<br>☐ Research in human<br>**Exclude**<br>☐ Descriptive studies (case reports, case studies), Sero-epidemiological studies, ecological or mathematical modelling studies
Population	**Include**<br>☐ Symptomatic infected individuals in the febrile phase **AND**<br>☐ Laboratory confirmed dengue diagnosis according to WHO Dengue Guideline in 1997 and 2009*<br>**Exclude**<br>☐ Individuals who had developed clinical manifestation of severe dengue (severe plasma leakage, hemodynamic instability and organ impairment)
Exposure(s)	**Include**<br>☐ Demographic features, clinical manifestations (signs and symptoms), laboratory parameters, or imaging techniques AND<br>☐ Parameters collected during the febrile phase<br>**Exclude**<br>☐ Genetic factors or gene expression
Outcome(s)	**Include**<br>☐ Association between predictors and risk of development severe clinical manifestations of dengue infection according to WHO guideline (dengue haemorrhagic fever grade I-IV, dengue shock syndrome, and severe dengue) compared with dengue fever

Overall:	☐ Include<br>☐ Exclude

Supplement Table 3: Template used for extracting information from the studies included in the systematic review.

**Paper ID**
**Title**
**Reviews**
**First author's surname**
**Year of publication**
**Study design**
**Data collection**
**Year of patient recruitment** (Month/Year - Month/year)
**Country** where patients were recruited
**Hospital/centre** names where patient were recruited
**Population** inclusion and exclusion criteria
**Dengue classification**<br>1: WHO 1997 (DS, DHF, and DSS)<br>2: WHO 2009 (DWS-, DWS+,and SD)
When were samples or information collected
**Reference group** (e.g. a DF group)
**Comparative group** (i.e. DHF, DSS, DHF/DSS, DWS+/SD, or SD)
**Continuous factors**_1 name (unit)
Description (if applicability)
How were samples or information measured (if applicable)
Sample size of a reference groups
Sample size of a comparative group
Ref: Mean, Median, Mean difference
Ref: SD, Upper 95% CI, Lower 95% CI, IQR, Upper IQR, Lower IQR
Ref: Odds ratio, Relative risk, Adjusted odds ration, Adjusted relative risk
Ref: 95% CI of OR
Comp: Mean, Median, Mean difference
Comp: SD, Upper 95% CI, Lower 95% CI, IQR, Upper IQR, Lower IQR
P-value
Statistic test
Comp: Odds ratio, Relative risk, Adjusted odds ratio, Adjusted relative risk
Comp: 95% CI of OR
P-value
Statistic test
Adjusted variables
**Categorical factors**_1 name (unit):
Description (if necessary)
How were samples or information measured (if necessary)
Sample size of a reference groups
Sample size of a comparative group

Level 1: The number of non-events (in reference group)
Level 1: The number of events (in comparison group)
Level 2: The number of non-events (in reference group)
Level 2: The number of events (in comparison group)
Level 3: The number of non-events (in reference group)
Level 3: The number of events (in comparison group)
P-value
Statistic test
Comp: Odds ratio, Relative risk, Adjusted odds ratio, Adjusted relative risk
Comp: Upper 95% CI
Comp: Lower 95% CI
Comp: Odds ratio, Relative risk, Adjusted odds ratio, Adjusted relative risk
Comp: Upper 95% CI
Comp: Lower 95% CI
P-value
Statistic test

Supplement Table 5. Definitions of progression to severe disease taken from the included studies in this systematic review and meta-analysis

Terminology	Definitions
Definition A (based on DHF in the 1997 WHO Classification)	Thrombocytopenia<br>- A platelet count less than 100,000 cells/micro litre
	and<br>Plasma leakage<br>- Haematocrit rising 20% from baseline haematocrit, haematocrit levels measured during the first 96 hours of illness, average haematocrit levels based on population data<br>OR<br>- Fluid accumulation by chest X-ray, ultrasonography, or physical examinations (depending on health facility and completeness of medical records)
	and

	Haemorrhagic tendencies<br><br>- Presence of spontaneous bleeding<br><br>OR<br><br>- Mucosal bleeding including positive tourniquet test
Definition B (based on DSS in the 1997 WHO Classification)	Shock<br><br>- Narrow pulse pressure less than 20 mmHg<br><br>OR<br><br>- Systolic blood pressure less than 90 mmHg (or hypotension in accordance with the sex and age specific reference of population data)<br><br>OR<br><br>- Poor tissue perfusion such as clammy skin, urine output less than 0.5 ml/kg/hr
Definition C (based on SD in the 2009 WHO Classification)	Severe plasma leakage<br><br>- Plasma leakage resulting in shock, defined as<br><br>- Narrow pulse pressure less than 20 mmHg<br><br>OR<br><br>- Systolic blood pressure less than 90 mmHg (or hypotension in accordance with the sex and age specific reference of population data)<br><br>OR<br><br>- Poor tissue perfusion such as clammy skin, urine output less than 0.5 ml/kg/hr<br><br>- Plasma leakage resulting in respiratory distress<br><br>- Evidence of pleural effusion or ascites<br><br>AND

	- Clinical indicators of respiratory distress such as tachypnoea or low oxygen saturations.
	OR<br><br>Severe clinical bleeding<br><br>- Bleeding in vital organs<br>- Spontaneous bleeding from a mucosal area with indication for blood transfusion
	OR<br><br>Severe organ involvement<br><br>- Aspartate aminotransferase (AST) level >1,000 units/litre,<br>- Alanine aminotransferase (ALT) level >1,000 units/litre,<br><br>- Serum creatinine level equal or more than 3 times above baseline<br><br>- Myocarditis<br><br>- Encephalitis

Supplement Table 6: Publications included in the systematic review and meta-analysis

Number	First author's name (Publication year)	Countries	Study design	Population age groups	Classification	Included in meta-analysis
**1**	Butthep et al.(2012)[1]	Thailand	prospective cohort	N/A	1997 WHO Classification	No
**2**	Hernandez et al.(2014)[2]	Mexico	case-control	Adults	1997 WHO Classification	Yes
**3**	Butthep et al.(2006)[3]	Thailand	prospective cohort	Children	1997 WHO Classification	No
**4**	Conroy et al.(2015)[4]	Columbia	nested case control or case-cohort	Mixed population	1997 WHO Classification	Yes
**5**	Wang et al.(2003)[5]	Taiwan	nested case control or case-cohort	DENV-3 infection	1997 WHO Classification	Yes
**6**	Voraphani et al.(2010)[6]	Thailand	prospective cohort	Children	1997 WHO Classification	Yes
**7**	Alexander et al.(2011)[7]	Thailand, Philippines, Vietnam, Malaysia, Nicaragua, Venezuela, and Brazil	prospective cohort	Mixed population	Intervention criteria	Yes
**8**	Yung et al.(2015)[8]	Singapore	prospective cohort	Adults	Both 1997 and 2009 WHO Classifications	Yes
**9**	Bongsebandhu et al.(2008)[9]	Thailand	prospective cohort	Children	1997 WHO Classification	Yes
**10**	Yong et al.(2017)[10]	Malaysia	nested case control or case-cohort	Adults	2009 WHO Classification	Yes
**11**	Vicente et al.(2017)[11]	Brazil	nested case control or case-cohort	Mixed population	The classification of Brazilian Ministry of Health	Yes
**12**	Vejchapipat et al.(2006)[12]	Thailand	prospective cohort	N/A	1997 WHO Classification	Yes
**13**	Vasanwala et al.(2014)[13]	Singapore	prospective cohort	Adults	1997 WHO Classification	Yes
**14**	Teixeira et al.(2015)[14]	Bazil	case-control	Mixed population	1997 WHO Classification	Yes
**15**	Tricou et al.(2011) [15]	Vietnam	retrospective cohort	Adults	1997 WHO Classification	Yes
**16**	Trairatvorakul et al.(2005)[16]	Thailand	case-control	Children	1997 WHO Classification	Yes
**17**	Rocha et al.(2017)[17]	Brazil	prospective cohort	Mixed population	2009 WHO Classification	Yes
**18**	Tang et al.(2017)[18]	Singapore	nested case control or case-cohort	Adults	1997 WHO Classification	Yes
**19**	Sreenivasan et al.(2018)[19]	India	prospective cohort	Children	2009 WHO Classification	Yes

**20**	Sosothikul et al.(2007)[20]	Thailand	prospective cohort	Children	1997 WHO Classification	Yes
**21**	Sirikutt et al.(2014)[21]	Thailand	retrospective cohort	Children	1997 WHO Classification	Yes
**22**	Restrepo et al.(2008)[22]	Columbia	case-control	Infants	N/A	Yes
**23**	Zain et al.(2016)[23]	Indonesia	case-control	Adults	2009 WHO Classification	No
**24**	Oliveira et al.(2017)[24]	Brazil	nested case control or case-cohort	N/A	1997 WHO Classification	Yes
**25**	Pang et al.(2015)[25]	Singapore	case-control	Matching with age, gender, laboratory, year of diagnosis, type of care at provisional site	Both 1997 and 2009 WHO Classifications	Yes
**26**	Sani et al.(2017)[26]	Malaysia	retrospective cohort	Adults	2009 WHO Classification	Yes
**27**	Pandey et al.(2015)[27]	India	case-control	Mixed population	2009 WHO Classification	Yes
**28**	Tuan et al.(2017)[28]	Vietnam	prospective cohort	Children	2009 WHO Classification	Yes
**29**	Maron et al.(2010)[29]	El Savador	case-control	Children	1997 WHO Classification	Yes
**30**	Mohammed et al.(2010)[30]	Puerto Rico	case-control	Mixed population	1997 WHO Classification	Yes
**31**	Mallhi et al.(2015)[31]	Malaysia	retrospective cohort	Adults	1997 WHO Classification	Yes
**32**	Lee et al.(2015)[32]	Taiwan	retrospective cohort	Adults	2009 WHO Classification	Yes
**33**	Lam et al.(2017)[33]	Vietnam	prospective cohort	Children	1997 WHO Classification	Yes
**34**	Mohamed et al.(2013)[34]	Yemen	retrospective cohort	Mixed population	1997 WHO Classification	Yes
**35**	Oishi et al.(2006)[35]	Philipines	prospective cohort	Children	1997 WHO Classification	Yes
**36**	Zhang et al.(2017)[36]	China	retrospective cohort	Adults	2009 WHO Classification	No
**37**	Wong et al.(2014)[37]	Singapore	prospective cohort	Adults	1997 WHO Classification	Yes
**38**	Wichmann et al.(2004)[38]	Thailand	retrospective cohort	Mixed population	1997 WHO Classification	Yes
**39**	Sharmin et al.(2013)[39]	Bangladesh	nested case control or case-cohort	Mixed population	1997 WHO Classification	No
**40**	Kurane et al.(1991) [40]	Thailand	case-control	Children	1997 WHO Classification	Yes
**41**	Kurane et al.(1993) [41]	Thailand	case-control	Children	1997 WHO Classification	Yes
**42**	Kuo et al.(2017) [42]	Taiwan	retrospective cohort	Adults	2009 WHO Classification	Yes
**43**	Kumar et al.(2012)[43]	Singapore	nested case control or case-cohort	Adults	1997 WHO Classification	Yes

**44**	Kulasinghe et al.(2016)[44]	Sri Lanka	prospective cohort	Children	1997 WHO Classification	Yes
**45**	Koraka et al.(2004) [45]	Indonesia	nested case control or case-cohort	Children	1997 WHO Classification	Yes
**46**	Malavige et al.(2013)[46]	Sri Lanka	prospective cohort	Adults	2009 WHO Classification	Yes
**47**	Romero et al.(2013)[47]	Brazil	cross-sectional study	Female aged 15 to 49 years	1997 WHO Classification	Yes
**48**	Libraty et al.(2002)[48]	Thailand	nested case control or case-cohort	DENV-2 infection	1997 WHO Classification	Yes
**49**	Liao et al.(2015)[49]	China	nested case control or case-cohort	Adults	2009 WHO Classification	Yes
**50**	Lee et al.(2008)[50]	Singapore	retrospective cohort	Adults	1997 WHO Classification	Yes
**51**	Lee et al.(2006)[51]	Taiwan	retrospective cohort	Mixed population	1997 WHO Classification	Yes
**52**	Kalayanarooj et al.(1997)[52]	Thailand	prospective cohort	Children	1997 WHO Classification	No
**53**	Kalayanarooj et al.(2005)[53]	Thailand	cross-sectional study	Children	1997 WHO Classification	No
**54**	Hoang et al.(2010)[54]	Vietnam	nested case control or case-cohort	Mixed population	1997 WHO Classification	No
**55**	Harris et al.(2000)[55]	Nicaragua	cross-sectional study	Mixed population	1997 WHO Classification	Yes
**56**	Hammond et al.(2005)[56]	Nicaragua	prospective cohort	Mixed population	1997 WHO Classification	No
**57**	Libraty et al.(2002)[57]	Thailand	nested case control or case-cohort	DENV-3 infection	1997 WHO Classification	Yes
**58**	Ha et al.(2011)[58]	Vietnam	case-control	Children	1997 WHO Classification	No
**59**	Green et al.(1999)[59]	Thailand	case-control	Children	1997 WHO Classification	No
**60**	Fried et al.(2010)[60]	Thailand	prospective cohort	Children	1997 WHO Classification	Yes
**61**	Fragnound et al.(2015)[61]	Cambodia and Columbia	nested case control or case-cohort	Mixed population	1997 WHO Classification	Yes
**62**	Flamand et al.(2017)[62]	French Guiana	cross-sectional study	Mixed population	2009 WHO Classification	Yes
**63**	Chen et al.(2015)[63]	Taiwan	retrospective cohort	Mixed population	2009 WHO Classification	Yes
**64**	Colbert et al.(2007)[64]	Nicaragua	prospective cohort	Children	1997 WHO Classification	Yes
**65**	Cui et al.(2016)[65]	Singapore	nested case control or case-cohort	Adults	1997 WHO Classification	Yes
**66**	Bur et al.(2016)[66]	Indonesia	prospective cohort	Adults	Both 1997 and 2009 WHO Classifications	Yes
**67**	Chaiyaratana et al.(2008)[67]	Thailand	prospective cohort	Children	1997 WHO Classification	No

**68**	Andries et al.(2016)[68]	Cambodia	nested case control or case-cohort	Children	2009 WHO Classification	Yes
**69**	Murgue et al.(2001)[69]	French Polynesia	nested case control or case-cohort	Children	1997 WHO Classification	Yes
**70**	Biswas et al.(2015)[70]	Nicaragua	prospective cohort	Children	Both 1997 and 2009 WHO Classifications	Yes
**71**	Carier et al.(2006)[71]	Venezuela	prospective cohort	N/A	1997 WHO Classification	No
**72**	Lee et al.(2012)[72]	Singapore	retrospective cohort	Mixed population	Both 1997 and 2009 WHO Classifications	No
**73**	Alagarasu et al.(2012)[73]	India	nested case control or case-cohort	Mixed population	Both 1997 and 2009 WHO Classifications	Yes
**74**	Rathakrishnan et al.(2014)[74]	Malasia	prospective cohort	Adults	2009 WHO Classification	Yes
**75**	Aung et al.(2013)[75]	Thailand	retrospective cohort	Adults	2009 WHO Classification	Yes
**76**	Bandyopadhyay et al.(2016)[76]	India	retrospective cohort	Adults	1997 WHO Classification	No
**77**	Bozza et al.(2008)[77]	Brazil	prospective cohort	Adults	Both 1997 and 2009 WHO Classifications	Yes
**78**	Brasier et al.(2012)[78]	Venezuela	prospective cohort	Mixed population	1997 WHO Classification	Yes
**79**	Carrasco et al.(2014)[79]	Singapore	retrospective cohort	Mixed population	2009 WHO Classification	No
**80**	Endy et al.(2004)[80]	Thailand	prospective cohort	Children	1997 WHO Classification	Yes
**81**	Fadilah et al.(1999)[81]	Malaysia	prospective cohort	Adults	1997 WHO Classification	Yes
**82**	Flores-Mendoza et al.(2017)[82]	Mexico	prospective cohort	Adults	1997 WHO Classification	Yes
**83**	Fox et al.(2011)[83]	Vietnam	prospective cohort	Mixed population	1997 WHO Classification	Yes
**84**	Furuta et al.(2012)[84]	Vietnam	case-control	Children	1997 WHO Classification	Yes
**85**	Gopal et al.(2017)[85]	India	case-control	Adults	2009 WHO Classification	Yes
**86**	Green et al.(1999)[86]	Thailand	nested case control or case-cohort	Children	1997 WHO Classification	Yes
**87**	Guerrero et al.(2013)[87]	Colombia	case-control	Adults	2009 WHO Classification	Yes
**88**	Hoffmeister et al.(2014)[88]	Germany	retrospective cohort	Adults	2009 WHO Classification	Yes
**89**	Juffrie et al.(2001)[89]	Indonesia	case-control	Children	1997 WHO Classification	No
**90**	Khan et al.(2010)[90]	Pakistan	cross-sectional study	Mixed population	1997 WHO Classification	No
**91**	Khan et al.(2007)[91]	Pakistan	cross-sectional study	Mixed population	1997 WHO Classification	Yes

**92**	Khan et al.(2013)[92]	Pakistan	prospective cohort	Mixed population	1997 WHO Classification	Yes
**93**	Kuo et al.(2008)[93]	Taiwan	retrospective cohort	Adults	1997 WHO Classification	Yes
**94**	Koraka et al.(2001)[94]	Indonesia	nested case control or case-cohort	Children	1997 WHO Classification	Yes
**95**	Lee et al.(2009)[95]	Singapore	retrospective cohort	Adults	1997 WHO Classification	Yes
**96**	Lye et al.(2009)[96]	Singapore	retrospective cohort	Adults	1997 WHO Classification	Yes
**97**	Mairuhu et al.(2005)[97]	Indonesia	prospective cohort	Children	1997 WHO Classification	Yes
**98**	Giraldo et al.(2011)[98]	Brazil	retrospective cohort	Children	Both 1997 and 2009 WHO Classifications	Yes
**99**	Singla et al.(2016)[99]	India	prospective cohort	Children	2009 WHO Classification	Yes
**100**	Vuong et al.(2016)[100]	Vietnam	prospective cohort	Mixed population	2009 WHO Classification	Yes
**101**	Pereira et al.(2018)[101]	India	cross-sectional study	Adults	2009 WHO Classification	Yes
**102**	Phakhounthong et al.(2018)[102]	Cambodia	retrospective cohort	Children	2009 WHO Classification	Yes
**103**	Mondragon et al.(2017)[103]	Mexico	nested case control or case-cohort	Adults	1997 WHO Classification	Yes
**104**	Pothapregada et al.(2015)[104]	India	cross-sectional study	Children	1997 WHO Classification	Yes
**105**	Potts et al.(2010)[105]	Thailand	retrospective cohort	Children	1997 WHO Classification	Yes
**106**	Raza et al.(2014)[106]	Pakistan	cross-sectional study	Mixed population	1997 WHO Classification	Yes
**107**	Sirivichayakul et al.(2012)[107]	Thailand	prospective cohort	Children	1997 WHO Classification	Yes
**108**	Sharmar et al.(2015)[108]	India	prospective cohort	Adults	1997 WHO Classification	Yes
**109**	Thein et al.(2015)[109]	Singapore	prospective cohort	Adults	1997 WHO Classification	Yes
**110**	Tissara et al.(2017)[110]	Sir Lanka	nested case control or case-cohort	Children	1997 WHO Classification	Yes
**111**	Thanachartwet et al.(2016)[111]	Thailand	prospective cohort	Adults	2009 WHO Classification	Yes
**112**	Thanacharwet et al.(2015)[112]	Thailand	prospective cohort	Adults	2009 WHO Classification	Yes
**113**	Thanachartwet et al.(2016)[113]	Thailand	prospective cohort	Adults	2009 WHO Classification	Yes
**114**	Thanachratwet et al.(2016)[114]	Thailand	prospective cohort	Adults	2009 WHO Classification	Yes
**115**	Vasanwala et al.(2011)[115]	Singapore	prospective cohort	Mixed population	1997 WHO Classification	Yes
**116**	Villar-Centrno et al.(2008)[116]	Colombia	prospective cohort	Mixed population	1997 WHO Classification	Yes

**117**	Garcia-Rivera et al.(2003)[117]	Puerto Rico	cross-sectional study	Mixed population	1997 WHO Classification	Yes
**118**	Prasad, D.; Bhriguvanshi, A. et al. (2020)[118]	India	cohort	children	2009 WHO Classification	Yes
**119**	Chandrashekhar et al. (2019) [119]	India	cohort	children	2009 WHO Classification	No
**120**	Elenga et al. (2019) [120]	French Guiana	cohort	children	2009 WHO Classification	No
**121**	Goncalves, B. S. et al. (2019) [121]	Brazil	cohort	mixed population	2009 WHO Classification	Yes
**122**	Goncalves, B. S. et al. (2) et al. (2019) [122]	Brazil	cohort	mixed population	2009 WHO Classification	Yes
**123**	Phuong, N. T. N. et al. (2019) [123]	Vietnam	cohort	children	2009 WHO Classification	Yes
**124**	Ta, T. V. at al. et al. (2019) [124]	Vietnam	cohort	children	1997 WHO Classification	Yes
**125**	May, W. L. et al. et al. (2019) [125]	Myanma	cross-sectional study	children	2009 WHO Classification	Yes
**126**	Nguyen Phung [126], N. T. et al. (2019)	Vietnam	cross-sectional study	children	2009 WHO Classification	No
**127**	Patra, Goutam et al. (2019) [127]	India	cohort	mixed population	2009 WHO Classification	Yes
**128**	Wang, W. H. et al. (2019) [128]	Taiwan	cohort	mixed population	1997 WHO Classification	Yes
**129**	Opasawatchai, A et al. (2019) [129]	Thailand	cohort	others	1997 WHO Classification	No
**130**	Kularatnam, G. A. M. et al (2019) [130]	Sri Lanka	cohort	children	1997 WHO Classification	Yes
**131**	Agrawal, V. K. et al. et al. (2018) [131]	India	cohort	mixed population	2009 WHO Classification	Yes
**132**	Athira, P. P. et al. (2018) [132]	India	cross-sectional study	children	2009 WHO Classification	No
**133**	Boillat-Blanco, N. et al. (2018) [133]	Tanzania	cohort	others	2009 WHO Classification	Yes
**134**	Lee, I. K. et al. (2018) [134]	Taiwan	cross-sectional study	others	2009 WHO Classification	Yes
**135**	Low, G. K. K. et al. (2018) [135]	Malaysia	cohort	others	2009 WHO Classification	Yes
**136**	Saniathi, N. K. E et al. (2018) [136]	Indonesia	case-control	children	1997 WHO Classification	Yes
**137**	Srivastava, G.; Chhavi, N.; Goel, A. (2018) [137]	India	cohort	children	2009 WHO Classification	Yes
**138**	Temprasertrudee, S. et al. (2018) [138]	Thailand	cohort	others	2009 WHO Classification	Yes
**139**	Villamor, E. et al. (2018) [139]	Colombia	case-control	mixed population	1997 WHO Classification	Yes
**140**	Wakimoto, M. D. et al. (2018) [140]	Brazil	case-control	children	2009 WHO Classification	Yes
**141**	Masood, Kiran Iqbal et al. (2018) [141]	Pakistan	case-control	mixed population	2009 WHO Classification	Yes
**142**	Wijeratne, D. T. et al. (2018) [142]	Colombia	cohort	others	1997 WHO Classification	Yes

**143**	Zhang, H. et al. (2018) [143]	China	cohort	others	2009 WHO Classification	Yes
**144**	Ralapanawa, U. et al. (2018) [144]	Sri Lanka	cross-sectional study	others	1997 WHO Classification	Yes
**145**	Mapalagamage, M. et al. (2018) [145]	Sri Lanka	cohort	others	1997 WHO Classification	Yes
**146**	Hegazi, M. A. et al. (2020) [146]	Saudiarabia	cohort	mixed population	2009 WHO Classification	Yes
**147**	Mapalagamage, M. et al. (2019) [147]	Sri Lanka	case-control	mixed population	2009 WHO Classification	No
**148**	Patra, G.; Saha, B.; Mukhopadhyay, S. (2019) [148]	India	cohort	mixed population	2009 WHO Classification	Yes
**149**	Sani, H. et al et al. (2019) [149]	Malaysia	cross-sectional study	others	2009 WHO Classification	Yes
**150**	Mahmud, Muhammad Rizwan et al. (2018) [150]	Pakistan	cohort	mixed population	1997 WHO Classification	Yes

References

1. Butthep P, Chunhakan S, Yoksan S, Tangnararatchakit K, Chuansumrit A. Alteration of cytokines and chemokines during febrile episodes associated with endothelial cell damage and plasma leakage in dengue hemorrhagic fever. Pediatric Infectious Disease Journal. 2012;31(12):e232-e8.

2. del Moral-Hernandez O, Martinez-Hernandez NE, Mosso-Pani MA, Hernandez-Sotelo D, Illades-Aguiar B, Flores-Alfaro E, et al. Association DENV1 and DENV2 infection with high serum levels of soluble thrombomodulin and VEGF in patients with dengue fever and dengue hemorrhagic fever. International Journal of Clinical and Experimental Medicine. 2014;7(2):370-8.

3. Butthep P, Chunhakan S, Tangnararatchakit K, Yoksan S, Pattanapanyasat K, Chuansumrit A. Elevated soluble thrombomodulin in the febrile stage related to patients at risk for dengue shock syndrome. Pediatric Infectious Disease Journal. 2006;25(10):894-7.

4. Conroy AL, Gelvez M, Hawkes M, Rajwans N, Tran V, Liles WC, et al. Host biomarkers are associated with progression to dengue haemorrhagic fever: A nested case-control study. International Journal of Infectious Diseases. 2015;40:45-53.

5. Wang WK, Chao DY, Kao CL, Wu HC, Liu YC, Li CM, et al. High levels of plasma dengue viral load during defervescence in patients with dengue hemorrhagic fever: Implications for pathogenesis. Virology. 2003;305(2):330-8.

6. Voraphani N, Theamboonlers A, Khongphatthanayothin A, Srisai C, Poovorawan Y. Increased level of hepatocyte growth factor in children with dengue virus infection. Annals of Tropical Paediatrics. 2010;30(3):213-8.

7. Alexander N, Balmaseda A, Coelho ICB, Dimaano E, Hien TT, Hung NT, et al. Multicentre prospective study on dengue classification in four South-east Asian and three Latin American countries. Tropical Medicine and International Health. 2011;16(8):936-48.

8. Yung CF, Lee KS, Thein TL, Tan LK, Gan VC, Wong JG, et al. Dengue serotype-specific differences in clinical manifestation, hematological parameters, plasma viral concentration and risk of severe disease in adults. American Journal of Tropical Medicine and Hygiene. 2013;1):35.

9. Bongsebandhu-Phubhakdi C, Hemungkorn M, Thisyakorn U, Thisyakorn C. Risk factors influencing severity in pediatric Dengue infection. Asian Biomedicine. 2008;2(5):409-13.

10. Yong YK, Tan HY, Jen SH, Shankar EM, Natkunam SK, Sathar J, et al. Aberrant monocyte responses predict and characterize dengue virus infection in individuals with severe disease. Journal of Translational Medicine. 2017;15 (1) (no pagination)(121).

11. Vicente CR, Cerutti Junior C, Froschl G, Romano CM, Cabidelle AS, Herbinger KH. Influence of demographics on clinical outcome of dengue: a cross-sectional study of 6703 confirmed cases in Vitoria, Espirito Santo State, Brazil. Epidemiol Infect. 2017;145(1):46-53.

12. Vejchapipat P, Theamboonlers A, Chongsrisawat V, Poovorawan Y. An evidence of intestinal mucosal injury in dengue infection. Southeast Asian Journal of Tropical Medicine and Public Health. 2006;37(1):79-82.

13. Vasanwala FF, Thein T, Leo Y, Gan VC, Hao Y, Lee LK, et al. Predictive value of proteinuria in adult dengue severity. PLoS Neglected Tropical Diseases. 2014;8(2).

14. Teixeira MG, Paixao ES, Costa MCN, Cunha RV, Pamplona L, Dias JP, et al. Arterial Hypertension and Skin Allergy Are Risk Factors for Progression from Dengue to Dengue Hemorrhagic Fever: A Case Control Study. PLoS Neglected Tropical Diseases. 2015;9 (5) (no pagination)(e0003812).

15. Tricou V, Nguyet Nguyen M, Farrar J, Hien Tinh T, Simmons CP. Kinetics of viremia and NS1 antigenemia are shaped by immune status and virus serotype in adults with dengue. PLoS Neglected Tropical Diseases. 2011;5(9).

16. Trairatvorakul P, Chongsrisawat V, Ngamvasinont D, Asawarachun D, Nantasook J, Poovorawan Y. Serum nitric oxide in children with dengue infection. Asian Pacific Journal of Allergy and Immunology. 2005;23(2-3):115-9.

17. Rocha BAM, Guilarde AO, Argolo AFLT, Tassara MP, da Silveira LA, Junqueira IC, et al. Dengue-specific serotype related to clinical severity during the 2012/2013 epidemic in centre of Brazil. Infectious Diseases of Poverty. 2017;6 (1) (no pagination)(116).

18. Tang TH, Alonso S, Ng LF, Thein TL, Pang VJ, Leo YS, et al. Increased Serum Hyaluronic Acid and Heparan Sulfate in Dengue Fever: Association with Plasma Leakage and Disease Severity. Scientific Reports. 2017;7:46191.

19. Sreenivasan P, S G, K S. Development of a Prognostic Prediction Model to Determine Severe Dengue in Children. Indian Journal of Pediatrics. 2018:1-7.

20. Sosothikul D, Seksarn P, Pongsewalak S, Thisyakorn U, Lusher J. Activation of endothelial cells, coagulation and fibrinolysis in children with Dengue virus infection. Thrombosis and Haemostasis. 2007;97(4):627-34.

21. Sirikutt P, Kalayanarooj S. Serum lactate and lactate dehydrogenase as parameters for the prediction of dengue severity. Journal of the Medical Association of Thailand. 2014;97(Supplement 6):S220-S31.

22. Restrepo BN, Isaza DM, Salazar CL, Ramirez R, Ospina M, Alvarez LG. Serum levels of interleukin-6, tumor necrosis factor-alpha and interferon-gamma in infants with and without dengue. Revista Da Sociedade Brasileira de Medicina Tropical. 2008;41(1):6-10.

23. Nurfadly Z, Suhartono TP, Umar Z, Herman H. Soluble Fas ligand as a potential marker of severity of dengue infection. Malaysian Journal of Medical Sciences. 2017;24(2):28-32.

24. Oliveira RADS, Cordeiro MT, Moura PMMFD, Baptista Filho PNB, Braga-Neto UDM, Marques ETDA, et al. Serum cytokine/chemokine profiles in patients with dengue fever (DF) and dengue hemorrhagic fever (FHD) by using protein array. Journal of Clinical Virology. 2017;89:39-45.

25. Pang JX, Thein TL, Lye DC, Leo YS. Differential Clinical Outcome of Dengue Infection among Patients with and without HIV Infection: A Matched Case Control Study. American Journal of Tropical Medicine and Hygiene. 2015;92(6):1156-62.

26. Md Sani SS, Han WH, Bujang MA, Ding HJ, Ng KL, Amir Shariffuddin MA. Evaluation of creatine kinase and liver enzymes in identification of severe dengue. BMC Infectious Diseases. 2017;17 (1) (no pagination)(505).

27. Pandey N, Jain A, Garg RK, Kumar R, Agrawal OP, Lakshmana Rao PV. Serum levels of IL-8, IFNgamma, IL-10, and TGF beta and their gene expression levels in severe and non-severe cases of dengue virus infection. Archives of virology. 2015;160(6):1463-75.

28. Nguyen MT, Ho TN, Nguyen VV, Nguyen TH, Ha MT, Ta VT, et al. An Evidence-Based Algorithm for Early Prognosis of Severe Dengue in the Outpatient Setting. Clinical Infectious Diseases. 2017;64(5):656-63.

29. Maron GM, Clara AW, Diddle JW, Pleites EB, Miller L, MacDonald G, et al. Association between nutritional status and severity of dengue infection in children in el salvador. American Journal of Tropical Medicine and Hygiene. 2010;82(2):324-9.

30. Mohammed H, Ramos M, Armstrong J, Munoz-Jordan J, Arnold-Lewis KO, Ayala A, et al. An outbreak of dengue fever in St. Croix (US Virgin Islands), 2005. PLoS ONE. 2010;32.

31. Mallhi TH, Khan AH, Adnan AS, Sarriff A, Khan YH, Jummaat F. Clinico-laboratory spectrum of dengue viral infection and risk factors associated with dengue hemorrhagic fever: A retrospective study. BMC Infectious Diseases. 2015;15 (1) (no pagination)(399).

32. Lee IK, Liu JW, Chen YH, Chen YC, Tsai CY, Huang SY, et al. Development of a simple clinical risk score for early prediction of severe dengue in adult patients. PLoS ONE. 2016;11 (5) (no pagination)(e0154772).

33. Lam PK, Ngoc TV, Thu Thuy TT, Hong Van NT, Nhu Thuy TT, Hoai Tam DT, et al. The value of daily platelet counts for predicting dengue shock syndrome: Results from a prospective observational study of 2301 Vietnamese children with dengue. PLoS Neglected Tropical Diseases. 2017;11 (4) (no pagination)(e0005498).

34. Mohamed NA, El-Raoof EA, Ibraheem HA. Respiratory manifestations of dengue fever in Taiz-Yemen. Egyptian Journal of Chest Diseases and Tuberculosis. 2013;62(2):319-23.

35. Oishi K, Mapua CA, Carlos CC, Cinco-Abanes MTDD, Saito M, Inoue S, et al. Dengue and other febrile illnesses among children in the Philippines. Dengue Bulletin. 2006;30:26-34.

36. Zhang H, Xie WZ, Xie SX, Ou YY, Zeng TW, Zhou PY. A novel predictor of patients with severe dengue: The aspartate aminotransferase/platelet count ratio index (APRI). Hepatology International. 2017;11 (1 Supplement 1):S548-S9.

37. Wong JG, Gan VC, Ng EL, Leo YS, Lye DC. Self-reported pain intensity using the numeric reporting scale in adult dengue management. American Journal of Tropical Medicine and Hygiene. 2013;1):180.

38. Wichmann O, Hongsiriwon S, Bowonwatanuwong C, Chotivanich K, Sukthana Y, Pukrittayakamee S. Risk factors and clinical features associated with severe dengue infection in adults and children during the 2001 epidemic in Chonburi, Thailand. Tropical Medicine and International Health. 2004;9(9):1022-9.

39. Sharmin R, Tabassum S, Mamun KZ, Nessa A, Jahan M. Dengue infection in Dhaka City, Bangladesh. Mymensingh medical journal : MMJ. 2013;22(4):781-6.

40. Kurane I, Innis BL, Nimmannitya S, Nisalak A, Meager A, Janus J, et al. Activation of T lymphocytes in dengue virus infections: High levels of soluble interleukin 2 receptor, soluble CD4, soluble CD8, interleukin 2, and interferon-gamma in sera of children with dengue. Journal of Clinical Investigation. 1991;88(5):1473-80.

41. Kurane I, Innis BL, Nimmannitya S, Nisalak A, Meager A, Ennis FA. High levels of interferon alpha in the sera of children with dengue virus infection. American Journal of Tropical Medicine and Hygiene. 1993;48(2):222-9.

42. Kuo HJ, Lee IK, Liu JW. Analyses of clinical and laboratory characteristics of dengue adults at their hospital presentations based on the World Health Organization clinical-phase framework: Emphasizing risk of severe dengue in the elderly. Journal of Microbiology, Immunology and Infection. 2017.

43. Kumar Y, Liang C, Bo Z, Rajapakse JC, Ooi EE, Tannenbaum SR. Serum Proteome and Cytokine Analysis in a Longitudinal Cohort of Adults with Primary Dengue Infection Reveals Predictive Markers of DHF. PLoS Neglected Tropical Diseases. 2012;6 (11) (no pagination)(e1887).

44. Kulasinghe S, Ediriweera R, Kumara P. Association of abnormal coagulation tests with dengue virus infection and their significance as early predictors of fluid leakage and bleeding. Sri Lanka Journalof Child Health. 2016;45(3):184-8.

45. Koraka P, Murgue B, Deparis X, Van Gorp ECM, Setiati TE, Osterhaus ADME, et al. Elevation of Soluble VCAM-1 Plasma Levels in Children with Acute Dengue Virus Infection of Varying Severity. Journal of Medical Virology. 2004;72(3):445-50.

46. Malavige GN, Gomes L, Alles L, Chang T, Salimi M, Fernando S, et al. Serum IL-10 as a marker of severe dengue infection. BMC Infectious Diseases. 2013;13 (1) (no pagination)(341).

47. Machado CR, Machado ES, Denis Rohloff R, Azevedo M, Campos DP, de Oliveira RB, et al. Is Pregnancy Associated with Severe Dengue? A Review of Data from the Rio de Janeiro Surveillance Information System. PLoS Neglected Tropical Diseases. 2013;7 (5) (no pagination)(e2217).

48. Libraty DH, Young PR, Pickering D, Endy TP, Kalayanarooj S, Green S, et al. High circulating levels of the dengue virus nonstructural protein NS1 early in dengue illness correlate with the development of dengue hemorrhagic fever. Journal of Infectious Diseases. 2002;186(8):1165-8.

49. Liao B, Tang Y, Hu F, Zhou W, Yao X, Hong W, et al. Serum levels of soluble vascular cell adhesion molecules may correlate with the severity of dengue virus-1 infection in adults. Emerging microbes & infections. 2015;4(4):e24.

50. Lee VJ, Lye DCB, Sun Y, Fernandez G, Ong A, Leo YS. Predictive value of simple clinical and laboratory variables for dengue hemorrhagic fever in adults. Journal of Clinical Virology. 2008;42(1):34-9.

51. Lee MS, Hwang KP, Chen TC, Lu PL, Chen TP. Clinical characteristics of dengue and dengue hemorrhagic fever in a medical center of southern Taiwan during the 2002 epidemic. Journal of Microbiology, Immunology and Infection. 2006;39(2):121-9.

52. Kalayanarooj S, Vaughn DW, Nimmannitya S, Green S, Suntayakorn S, Kunentrasai N, et al. Early clinical and laboratory indicators of acute dengue illness. Journal of Infectious Diseases. 1997;176(2):313-21.

53. Kalayanarooj S, Nimmannitya S. Is dengue severity related to nutritional status? Southeast Asian Journal of Tropical Medicine and Public Health. 2005;36(2):378-84.

54. Hoang LT, Lynn DJ, Henn M, Birren BW, Lennon NJ, Le PT, et al. The early whole-blood transcriptional signature of dengue virus and features associated with progression to dengue shock syndrome in Vietnamese children and young adults. Journal of Virology. 2010;84(24):12982-94.

55. Harris E, Videa E, Perez L, Sandoval E, Tellez Y, Perez MD, et al. Clinical, epidemiologic, and virologic features of dengue in the 1998 epidemic in Nicaragua. American Journal of Tropical Medicine and Hygiene. 2000;63(1-2):5-11.

56. Hammond SN, Balmaseda A, Perez L, Tellez Y, Saborio SI, Mercado JC, et al. Differences in dengue severity in infants, children, and adults in a 3-year hospital-based study in Nicaragua. American Journal of Tropical Medicine and Hygiene. 2005;73(6):1063-70.

57. Libraty DH, Endy TP, Houng HS, Green S, Kalayanarooj S, Suntayakorn S, et al. Differing influences of virus burden and immune activation on disease severity in secondary dengue-3 virus infections. Journal of Infectious Diseases. 2002;185(9):1213-21.

58. Ha TTN, Huy NT, Murao LA, Lan NTP, Thuy TT, Tuan HM, et al. Elevated levels of cell-free circulating DNA in patients with acute dengue virus infection. PLoS ONE. 2011;6 (10) (no pagination)(e25969).

59. Green S, Pichyangkul S, Vaughn DW, Kalayanarooj S, Nimmannitya S, Nisalak A, et al. Early CD69 expression on peripheral blood lymphocytes from children with dengue hemorrhagic fever. Journal of Infectious Diseases. 1999;180(5):1429-35.

60. Fried JR, Gibbons RV, Kalayanarooj S, Thomas SJ, Srikiatkhachorn A, Yoon IK, et al. Serotype-specific differences in the risk of dengue hemorrhagic fever: An analysis of data collected in Bangkok, Thailand from 1994 to 2006. PLoS Neglected Tropical Diseases. 2010;4 (3) (no pagination)(e617).

61. Fragnoud R, Flamand M, Reynier F, Buchy P, Duong V, Pachot A, et al. Differential proteomic analysis of virus-enriched fractions obtained from plasma pools of patients with dengue fever or severe dengue. BMC Infectious Diseases. 2015;15 (1) (no pagination)(518).

62. Flamand C, Fritzell C, Prince C, Abboud P, Ardillon V, Carvalho L, et al. Epidemiological assessment of the severity of dengue epidemics in French Guiana. Plos One. 2017;12(2).

63. Chen C-C, Lee I-K, Liu J-W, Huang S-Y, Wang L. Utility of C-Reactive Protein Levels for Early Prediction of Dengue Severity in Adults. BioMed Research International. 2015;2015:1-6.

64. Colbert JA, Gordon A, Roxelin R, Silva S, Silva J, Rocha C, et al. Ultrasound measurement of gallbladder wall thickening as a diagnostic test and prognostic indicator for severe dengue in pediatric patients. Pediatric Infectious Disease Journal. 2007;26(9):850-2.

65. Cui L, Lee YH, Thein TL, Fang J, Pang J, Ooi EE, et al. Serum Metabolomics Reveals Serotonin as a Predictor of Severe Dengue in the Early Phase of Dengue Fever. PLoS Neglected Tropical Diseases. 2016;10 (4) (no pagination)(e0004607).

66. Bur R, Suwarto S, Santoso WD, Harimurti K. Serum lactate as predictor and diagnostic biomarker of plasma leakage in adult dengue patients. Universa Medicina. 2016;35(3):213-21.

67. Chaiyaratana W, Chuansumrit A, Atamasirikul K, Tangnararatchakit K. Serum ferritin levels in children with dengue infection. The Southeast Asian journal of tropical medicine and public health. 2008;39(5):832-6.

68. Andries AC, Duong V, Cappelle J, Ong S, Kerleguer A, Ly S, et al. Proteinuria during dengue fever in children. International Journal of Infectious Diseases. 2017;55:38-44.

69. Murgue B, Cassar O, Deparis X. Plasma concentrations of sVCAM-1 and severity of dengue infections. Journal of Medical Virology. 2001;65(1):97-104.

70. Biswas H, Gordon A, Elizondo D, Nunez A, Rocha C, Perez MA, et al. Lower cholesterol levels are associated with severe dengue outcome. American Journal of Tropical Medicine and Hygiene. 2014;1):57.

71. Cardier JE, Balogh V, Perez-Silva C, Romano E, Rivas B, Bosch N, et al. Relationship of thrombopoietin and interleukin-11 levels to thrombocytopenia associated with dengue disease. Cytokine. 2006;34(3-4):155-60.

72. Lee LK, Gan VC, Lee VJ, Tan AS, Leo YS, Lye DC. Clinical relevance and discriminatory value of elevated liver aminotransferase levels for dengue severity. PLoS Negl Trop Dis. 2012;6(6):e1676.

73. Alagarasu K, Bachal RV, Bhagat AB, Shah PS, Dayaraj C. Elevated levels of vitamin D and deficiency of mannose binding lectin in dengue hemorrhagic fever. Virology Journal. 2012;9.

74. Rathakrishnan A, Klekamp B, Wang SM, Komarasamy TV, Natkunam SK, Sathar J, et al. Clinical and immunological markers of dengue progression in a study cohort from a hyperendemic area in Malaysia. PLoS ONE. 2014;9 (3) (no pagination)(e92021).

75. Aung KL, Thanachartwet V, Desakorn V, Chamnanchanunt S, Sahassananda D, Chierakul W, et al. Factors associated with severe clinical manifestation of dengue among adults in Thailand. The Southeast Asian journal of tropical medicine and public health. 2013;44(4):602-12.

76. Bandyopadhyay D, Chattaraj S, Hajra A, Mukhopadhyay S, Ganesan V. A study on spectrum of hepatobiliary dysfunctions and pattern of liver involvement in dengue infection. Journal of Clinical and Diagnostic Research. 2016;10(5):OC21-OC6.

77. Bozza FA, Cruz OG, Zagne SMO, Azeredo EL, Nogueira RMR, Assis EF, et al. Multiplex cytokine profile from dengue patients: MIP-1beta and IFN-gamma as predictive factors for severity. BMC Infectious Diseases. 2008;8 (no pagination)(86).

78. Brasier AR, Ju H, Garcia J, Spratt HM, Victor SS, Forshey BM, et al. A three-component biomarker panel for prediction of dengue hemorrhagic fever. American Journal of Tropical Medicine and Hygiene. 2012;86(2):341-8.

79. Carrasco LR, Leo YS, Cook AR, Lee VJ, Thein TL, Go CJ, et al. Predictive Tools for Severe Dengue Conforming to World Health Organization 2009 Criteria. PLoS Neglected Tropical Diseases. 2014;8 (7) (no pagination)(e2972).

80. Endy TP, Nisalak A, Chunsuttitwat S, Vaughn DW, Green S, Ennis FA, et al. Relationship of preexisting dengue virus (DV) neutralizing antibody levels to viremia and severity of disease in a prospective cohort study of DV infection in Thailand. Journal of Infectious Diseases. 2004;189(6):990-1000.

81. Fadilah SA, Sahrir S, Raymond AA, Cheong SK, Aziz JA, Sivagengei K. Quantitation of T lymphocyte subsets helps to distinguish dengue hemorrhagic fever from classic dengue fever during the acute febrile stage. The Southeast Asian journal of tropical medicine and public health. 1999;30(4):710-7.

82. Flores-Mendoza LK, Estrada-Jimenez T, Sedeno-Monge V, Moreno M, Manjarrez MDC, Gonzalez-Ochoa G, et al. IL-10 and socs3 Are Predictive Biomarkers of Dengue Hemorrhagic Fever. Mediators of Inflammation. 2017;2017:5197592.

83. Fox A, Hoa LNM, Simmons CP, Wolbers M, Wertheim HFL, Khuong PT, et al. Immunological and Viral Determinants of Dengue Severity in Hospitalized Adults in Ha Noi, Viet Nam. Plos Neglected Tropical Diseases. 2011;5(3).

84. Furuta T, Murao LA, Lan NTP, Huy NT, Huong VTQ, Thuy TT, et al. Association of mast cell-derived VEGF and proteases in dengue shock syndrome. PLoS Neglected Tropical Diseases. 2012;6 (2) (no pagination)(e1505).

85. Gopal SSS, Pillai AB, Ramachandrappaa VS, Kadhiravan T, Dhodapkar R, Kah J, et al. Increased serum levels of macrophage activation marker sCD163 in Dengue patients. Journal of Clinical Virology. 2017;86:62-7.

86. Green S, Vaughn DW, Kalayanarooj S, Nimmannitya S, Suntayakorn S, Nisalak A, et al. Early immune activation in acute dengue illness is related to development of plasma leakage and disease severity. Journal of Infectious Diseases. 1999;179(4):755-62.

87. Guerrero CD, Arrieta AF, Ramirez ND, Rodriguez LS, Vega R, Bosch I, et al. High plasma levels of soluble ST2 but not its ligand IL-33 is associated with severe forms of pediatric dengue. Cytokine. 2013;61(3):766-71.

88. Hoffmeister B, Suttorp N, Zoller T. The revised dengue fever classification in German travelers: clinical manifestations and indicators for severe disease. Infection. 2014;25.

89. Juffrie M, Vo Meer GM, Hack CE, Haasnoot K, Sutaryo, Veerman AJP, et al. Inflammatory mediators in dengue virus infection in children: Interleukin-6 and its relation to C-reactive protein and secretory phospholipase A2. American Journal of Tropical Medicine and Hygiene. 2001;65(1):70-5.

90. Khan E, Kisat M, Khan N, Nasir A, Ayub S, Hasan R. Demographic and clinical features of dengue fever in Pakistan from 2003-2007: A retrospective cross- sectional study. PLoS ONE. 2010;5(9):1-7.

91. Khan E, Siddiqui J, Shakoor S, Mehraj V, Jamil B, Hasan R. Dengue outbreak in Karachi, Pakistan, 2006: experience at a tertiary care center. Transactions of the Royal Society of Tropical Medicine and Hygiene. 2007;101(11):1114-9.

92. Khan MIH, Anwar E, Agha A, Hassanien NSM, Ullah E, Syed IA, et al. Factors predicting severe dengue in patients with dengue fever. Mediterranean Journal of Hematology and Infectious Diseases. 2013;5 (1) (no pagination)(e2013014).

93. Kuo MC, Lu PL, Chang JM, Lin MY, Tsai JJ, Chen YH, et al. Impact of renal failure on the outcome of dengue viral infection. Clinical Journal of the American Society of Nephrology. 2008;3(5):1350-6.

94. Koraka P, Suharti C, Setiati TE, Mairuhu ATA, Van Gorp E, Hach CE, et al. Kinetics of dengue virus-specific serum immunoglobulin classes and subclasses correlate with clinical outcome of infection. Journal of Clinical Microbiology. 2001;39(12):4332-8.

95. Lee VJ, Lye DC, Sun Y, Leo YS. Decision tree algorithm in deciding hospitalization for adult patients with dengue haemorrhagic fever in Singapore. Tropical Medicine and International Health. 2009;14(9):1154-9.

96. Lye DC, Lee VJ, Sun Y, Leo YS. The benign nature of acute dengue infection in hospitalized older adults in Singapore. International Journal of Infectious Diseases.

97. Mairuhu ATA, Peri G, Setiati TE, Hack CE, Koraka P, Soemantri A, et al. Elevated plasma levels of the long pentraxin, pentraxin 3, in severe dengue virus infections. Journal of Medical Virology. 2005;76(4):547-52.

98. Giraldo D, Sant'Anna C, Perisse AR, March Mde F, Souza AP, Mendes A, et al. Characteristics of children hospitalized with dengue fever in an outbreak in Rio de Janeiro, Brazil. Trans R Soc Trop Med Hyg. 2011;105(10):601-3.

99. Singla M, Kar M, Sethi T, Kabra SK, Lodha R, Chandele A, et al. Immune Response to Dengue Virus Infection in Pediatric Patients in New Delhi, India-Association of Viremia, Inflammatory Mediators and Monocytes with Disease Severity. PLoS Neglected Tropical Diseases. 2016;10 (3) (no pagination)(e0004497).

100. Vuong NL, Manh DH, Mai NT, Phuc LH, Luong VT, Quan VD, et al. Criteria of "persistent vomiting" in the WHO 2009 warning signs for dengue case classification. Tropical Medicine and Health. 2016;44 (1) (no pagination)(14).

101. Pereira MS, Kudru CU, Nair S, Thunga G, Kunhikatta V, Guddattu V. Factors associated with severity of illness in patients with dengue fever in a tertiary care hospital in southern India. Asian Journal of Pharmaceutical and Clinical Research. 2018;11(3):272-6.

102. Phakhounthong K, Chaovalit P, Jittamala P, Blacksell SD, Carter MJ, Turner P, et al. Predicting the severity of dengue fever in children on admission based on clinical features and laboratory indicators: Application of classification tree analysis. BMC Pediatrics. 2018;18 (1) (no pagination)(109).

103. Posadas-Mondragon A, Aguilar-Faisal JL, Chavez-Negrete A, Guillen-Salomon E, Alcantara-Farfan V, Luna-Rojas L, et al. Indices of anti-dengue immunoglobulin G subclasses in adult Mexican patients with febrile and hemorrhagic dengue in the acute phase. Microbiology and Immunology. 2017;61(10):433-41.

104. Pothapregada S, Kamalakannan B, Thulasingham M. Risk factors for shock in children with dengue fever. Indian Journal of Critical Care Medicine. 2015;19(11):661-4.

105. Potts JA, Thomas SJ, Srikiatkhachorn A, Supradish PO, Li W, Nisalak A, et al. Classification of dengue illness based on readily available laboratory data. Am J Trop Med Hyg. 2010;83(4):781-8.

106. Raza FA, Shafiq ur R, Ruqyya K, Jameel A, Ashraf S, Mazhar I, et al. Demographic and clinico-epidemiological features of dengue fever in Faisalabad, Pakistan. PLoS ONE. 2014;9(3).

107. Sirivichayakul C, Limkittikul K, Chanthavanich P, Jiwariyavej V, Chokejindachai W, Pengsaa K, et al. Dengue Infection in Children in Ratchaburi, Thailand: A Cohort Study. II. Clinical Manifestations. Plos Neglected Tropical Diseases. 2012;6(2).

108. Sharma K, Yadav A. Association of mean platelet volume with severity, serology & treatment outcome in dengue fever: Prognostic utility. Journal of Clinical and Diagnostic Research. 2015;9(11):EC01-EC3.

109. Thein TL, Wong J, Leo YS, Ooi EE, Lye D, Yeo TW. Association between increased vascular nitric oxide bioavailability and progression to dengue hemorrhagic fever in adults. Journal of Infectious Diseases. 2015;212(5):711-4.

110. Tissera H, Rathore APS, Wei Yee L, Pike BL, Warkentien TE, Farouk FS, et al. Chymase Level Is a Predictive Biomarker of Dengue Hemorrhagic Fever in Pediatric and Adult Patients. Journal of Infectious Diseases. 2017;216(9):1112-21.

111. Thanachartwet V, Desakorn V, Sahassananda D, Jittmittraphap A, Oer-areemitr N, Osothsomboon S, et al. Serum Procalcitonin and Peripheral Venous Lactate for Predicting Dengue Shock and/or Organ Failure: A Prospective Observational Study. PLoS Neglected Tropical Diseases. 2016;10 (8) (no pagination)(e0004961).

112. Thanachartwet V, Oer-areemitr N, Chamnanchanunt S, Sahassananda D, Jittmittraphap A, Suwannakudt P, et al. Identification of clinical factors associated with severe dengue among Thai adults: A prospective study. BMC Infectious Diseases. 2015;15(1).

113. Thanachartwet V, Wattanathum A, Oer-areemitr N, Jittmittraphap A, Sahassananda D, Monpassorn C, et al. Diagnostic accuracy of peripheral venous lactate and the 2009 WHO warning signs for identifying severe dengue in Thai adults: A prospective observational study. BMC Infectious Diseases. 2016;16 (1) (no pagination)(46).

114. Thanachartwet V, Wattanathum A, Sahassananda D, Wacharasint P, Chamnanchanunt S, Kyaw EK, et al. Dynamic Measurement of Hemodynamic Parameters and Cardiac Preload in Adults with Dengue: A Prospective Observational Study. Plos One. 2016;11(5).

115. Vasanwala FF, Puvanendran R, Fook-Chong S, Ng JM, Suhail SM, Lee KH. Could peak proteinuria determine whether patient with dengue fever develop dengue hemorrhagic/dengue shock syndrome?--a prospective cohort study. BMC Infectious Diseases. 2011;11:212.

116. Villar-Centeno LA, Díaz-Quijano FA, Martínez-Vega RA. Biochemical alterations as markers of dengue hemorrhagic fever. American Journal of Tropical Medicine and Hygiene. 2008;78(3):370-4.

117. García-Rivera EJ, Rigau-Pérez JG. Dengue severity in the elderly in Puerto Rico. Revista Panamericana de Salud Publica. 2003;13(6):362-8.

118. Prasad D, Bhriguvanshi A. Clinical Profile, Liver Dysfunction and Outcome of Dengue Infection in Children: A Prospective Observational Study. The Pediatric infectious disease journal. 2019;02.

119. Chandrashekhar C, Balaji K, Vasudev PH, Panachiyil GM, Babu T. Estimation of serum neopterin level as an early marker for detecting severe dengue infection. Int J Pediatr Adolesc Med. 2019;6(4):151-4.

120. Elenga N, Celicourt D, Muanza B, Elana G, Hocquelet S, Tarer V, et al. Dengue in hospitalized children with sickle cell disease: A retrospective cohort study in the French departments of America. Journal of Infection and Public Health. 2019.

121. Goncalves BS, Nogueira RMR, Bispo de Filippis AM, Horta MAP. Factors predicting the severity of dengue in patients with warning signs in Rio de Janeiro, Brazil (1986-2012). Trans R Soc Trop Med Hyg. 2019;113(11):670-7.

122. Goncalves BS, Horta MAP, Acero PHC, Bochner R, Queiroz Lima MDR, de Araujo ES, et al. Dynamics of nonstructural glycoprotein-1 in dengue patients presenting with different clinical manifestations from 1986 to 2012 in Rio de Janeiro, Brazil. Journal of Medical Virology. 2019;91(4):555-63.

123. Phuong NTN, Manh DH, Dumre SP, Mizukami S, Weiss LN, Van Thuong N, et al. Plasma cell-free DNA: a potential biomarker for early prediction of severe dengue. Ann Clin Microbiol Antimicrob. 2019;18(1):10.

124. Ta TV, Tran HT, Ha ONT, Nguyen XT, Tran VK, Pham HT, et al. The correlation of clinical and subclinical presentations with dengue serotypes and plasma viral load: The case of children with dengue hemorrhagic fever in Vietnam. International Journal of Research in Pharmaceutical Sciences. 2019;10(3):2578-85.

125. May WL, Kyaw MP, Blacksell SD, Pukrittayakamee S, Chotivanich K, Hanboonkunupakarn B, et al. Impact of glucose-6-phosphate dehydrogenase deficiency on dengue infection in Myanmar children. PLoS ONE. 2019;14 (1) (no pagination)(e0209204).

126. Nguyen Phung NT, Nguyen TTP, Bui TL, Nguyen TH, Tran DT. C-reactive protein in children with dengue fever in Vietnam. International Journal of Research in Pharmaceutical Sciences. 2019;10(3):2525-31.

127. Patra G, Mallik S, Saha B, Mukhopadhyay S. Assessment of chemokine and cytokine signatures in patients with dengue infection: A hospital-based study in Kolkata, India. Acta Tropica. 2019;190:73-9.

128. Wang WH, Lin CY, Chang K, Urbina AN, Assavalapsakul W, Thitithanyanont A, et al. A clinical and epidemiological survey of the largest dengue outbreak in Southern Taiwan in 2015. International Journal of Infectious Diseases. 2019;88:88-99.

129. Opasawatchai A, Amornsupawat P, Jiravejchakul N, Chan-In W, Spoerk NJ, Manopwisedjaroen K, et al. Neutrophil Activation and Early Features of NET Formation Are Associated With Dengue Virus Infection in Human. Frontiers in Immunology. 2018;9:3007.

130. Kularatnam GAM, Jasinge E, Gunasena S, Samaranayake D, Senanayake MP, Wickramasinghe VP. Evaluation of biochemical and haematological changes in dengue fever and dengue hemorrhagic fever in Sri Lankan children: a prospective follow up study. BMC Pediatrics. 2019;19(1):87.

131. Agrawal VK, Saroj Kumar Prusty B, Santosh Reddy C, Reddy GKM, Agrawal RK, Bandaru VCSS. Clinical profile and predictors of Severe Dengue disease: A study from South India. Caspian j. 2018;9(4):334-40.

132. Athira PP, Jagan OA, Umadevi P, Pragnatha K, Veena PM. A Retrospective Study of Paediatric Dengue Cases in a Tertiary Care Hospital in Southern India. Journal of Clinical and Diagnostic Research. 2018;12(7):SC01-SC6.

133. Boillat-Blanco N, Klaassen B, Mbarack Z, Samaka J, Mlaganile T, Masimba J, et al. Dengue fever in Dar es Salaam, Tanzania: clinical features and outcome in populations of black and non-black racial category. BMC Infectious Diseases. 2018;18(1):644.

134. Lee IK, Huang CH, Huang WC, Chen YC, Tsai CY, Chang K, et al. Prognostic Factors in Adult Patients with Dengue: Developing Risk Scoring Models and Emphasizing Factors Associated with Death <=7 Days after Illness Onset and <=3 Days after Presentation. Journal of Clinical Medicine. 2018;7(11):28.

135. Low GKK, Papapreponis P, Isa RM, Gan SC, Chee HY, Te KK, et al. Geographical distribution and spatio-temporal patterns of hospitalization due to dengue infection at a leading specialist hospital in Malaysia. Geospatial Health. 2018;13(1):127-34.

136. Saniathi NKE, Rianto BUD, Juffrie M, Soetjiningsih. The effect of overnutrition toward the risk of dengue shock syndrome in pediatric patient: in-depth investigation of sVCAM-1 and adiponectin level. Bali Medical Journal. 2018;7(1):244-8.

137. Srivastava G, Chhavi N, Goel A. Validation of Serum Aminotransferases Levels to Define Severe Dengue Fever in Children. Pediatr. 2018;21(4):289-96.

138. Temprasertrudee S, Thanachartwet V, Desakorn V, Keatkla J, Chantratita W, Kiertiburanakul S. A Multicenter Study of Clinical Presentations and Predictive Factors for Severe Manifestation of Dengue in Adults. Japanese Journal of Infectious Diseases. 2018;71(3):239-43.

139. Villamor E, Villar LA, Lozano-Parra A, Herrera VM, Herrán OF. Serum fatty acids and progression from dengue fever to dengue haemorrhagic fever/dengue shock syndrome. Br J Nutr. 2018;120(7):787-96.

140. Wakimoto MD, Camacho LAB, Gonin ML, Brasil P. Clinical and Laboratory Factors Associated with Severe Dengue: A Case-Control Study of Hospitalized Children. Journal of Tropical Pediatrics. 2018;64(5):373-81.

141. Masood KI, Jamil B, Rahim M, Islam M, Farhan M, Hasan Z. Role of TNF α, IL-6 and CXCL10 in Dengue disease severity. Iranian Journal of Microbiology. 2018;10(3):202-7.

142. Wijeratne DT, Fernando S, Gomes L, Jeewandara C, Ginneliya A, Samarasekara S, et al. Quantification of dengue virus specific T cell responses and correlation with viral load and clinical disease severity in acute dengue infection. Plos Neglected Tropical Diseases. 2018;12(10):16.

143. Zhang H, Xie ZW, Xie XS, Ou YY, Zeng WT, Zhou YP. A novel predictor of severe dengue: The aspartate aminotransferase/platelet count ratio index (APRI). Journal of Medical Virology. 2018;90(5):803-9.

144. Ralapanawa U, Alawattegama ATM, Gunrathne M, Tennakoon S, Kularatne SAM, Jayalath T. Value of peripheral blood count for dengue severity prediction. BMC Research Notes. 2018;11(1):400.

145. Mapalagamage M, Handunnetti S, Premawansa G, Thillainathan S, Fernando T, Kanapathippillai K, et al. Is Total Serum Nitrite and Nitrate (NOx) Level in Dengue Patients a Potential Prognostic Marker of Dengue Hemorrhagic Fever? Dis Markers. 2018;2018:5328681.

146. Hegazi MA, Bakarman MA, Alahmadi TS, Butt NS, Alqahtani AM, Aljedaani BS, et al. Risk Factors and Predictors of Severe Dengue in Saudi Population in Jeddah, Western Saudi Arabia: A Retrospective Study. Am J Trop Med Hyg. 2020;13:13.

147. Mapalagamage M, Handunnetti SM, Wickremasinghe AR, Premawansa G, Thillainathan S, Fernando T, et al. High levels of serum Angiopoietin 2 and Angiopoietin 2/1 ratio at critical stage of dengue hemorrhagic fever patients and their association with clinical and biochemical parameters. Journal of clinical microbiology. 2020;15.

148. Patra G, Saha B, Mukhopadhyay S. Study of serum VEGF levels in patients with severe dengue infection admitted in a tertiary care hospital in Kolkata. Journal of Medical Virology. 2019;91(10):1873-6.

149. Sani H, Syazana N, Izuan Z, Isa MR, Hanis M, Yazli M. Procalcitonin and clinical factors associated with severe dengue infection in hospitalized adults in Malaysia. American Journal of Infectious Diseases. 2019;15(4):103-10.

150. Mahmud MR, Zaman S, Naseem N, Iqbal N, Tanveer N, Khalid MA, et al. Comparison of Vitamin D Levels in Patients with Dengue Haemorrhagic Fever and Dengue Fever. Journal of Rawalpindi Medical College. 2018;22(2):92-5.

Supplement Table 7. Definitions of associated factors taken from the included studies in this systematic review and meta-analysis

Associated factors	Definition
Age	Age of participants in year or month units according to medical records or history taking
Sex	Physical sex according to medical records or history taking
Nutritional status	For children (≤ 18 years old), weight for age based on local standardized guidelines or BMI-for-age >2 Z score according to WHO sex-specific growth reference for children.<br><br>For adults (>18 years old) the BMI criterion was used, malnourished BMI < 18 and obese BMI > 30 kg/m^2
Weight	Weight of patients measured at presenting at hospitals or medical records
Mixed comorbidity	Unspecific pre-existing comorbidities including cancer, asthma, chronic obstructive pulmonary disease, etc. according to medical records or history taking
Hypertension	Pre-existing hypertension disease according to medical records or history taking
Diabetes mellitus	Pre-existing diabetes mellitus according to medical records or history taking

Renal disease	Pre-existing renal diseases excepting acute kidney injury on dengue episode according to medical records or history taking
Cardiovascular disease	Pre-existing cardiovascular disease including chronic heart failure, myocardial infarction according to medical records or history taking
Rash	Presence of unspecific rash during the first four days of the illness according to history taking or physical examinations
Vomiting	Presence of vomiting with any episodes during the first four days of the illness according to history taking or physical examinations
Abdominal pain and tenderness	Presence of abdominal pain and tenderness with any episodes during the first four days of the illness according to history taking or physical examinations
Headache	Presence of headache during the first four days of illness according to history taking
Minor bleeding	Presence of either mucosal or spontaneous bleeding that do not require blood transfusion and present during the first four days of illness. This includes skin bleeding (petechiae, ecchymosis, or purpura), mucosal bleeding (epistaxis, gum bleeding, or other sites), haematemesis or melena.
Positive tourniquet test	Tourniquet test with the presence of ≥20 petechiae per square inch.

Immune status	Secondary infection determined with the use of serological tests (i.e. either IgM or IgG ELISA during the first four days of illness or the convalescence phase.)
Clinical fluid accumulation	Presence of fluid accumulation (i.e. either pleural effusion or ascites) during the first four days of illness according to chest X-ray, ultrasonography, or physical examination
Serotypes	Infecting dengue serotypes determined using patients' serum collected during the first four days of illness with conventional or real-time RT-PCR assays, or viral isolation by mosquito inoculation.
Viraemia levels	Viral RNA levels were quantified using real-time PRC or quantitative RT-PCR assays in the patients' plasma specimens collected during the first four days of illness
Platelet count	The number of platelets during the first four days of illness. If there are reports of platelet count for more than one day, platelet count on day 3 of illness was chosen.
Leukocyte cell count	The number of leukocytes during the first four days of illness. If there are reports of leukocyte count for more than one day, leukocyte count on day 3 of illness was chosen.
Haematocrit	Haematocrit detected during the first four days of illness. If there are reports of haematocrit for more than one day, haematocrit on day 3 of illness was chosen.

Aspartate transaminase (AST)	AST levels detected during the first four days of illness. If there are reports of AST on more than one day, AST on day 3 of illness was chosen.
Alanine transaminase (ALT)	ALT levels detected during the first four days of illness. If there are reports of ALT on more than one day, ALT on day 3 of illness was chosen.
Serum albumin	Serum albumin levels detected during the first four days of illness. If there are reports of serum albumin on more than one day, albumin levels on day 3 of illness was chosen.

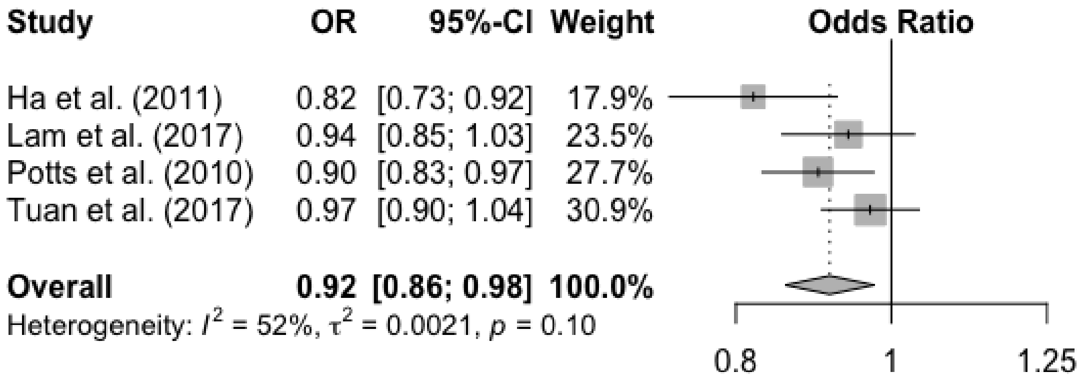

Supplement Figure 1: Forest plot of associations from multivariable models between age and severe progression. Four studies conducted among children (≤ 18 years old) were included in the meta-analysis. The pooled OR was 0.92 (95% CI (0.86, 0.98)) and I^2 was 52%.

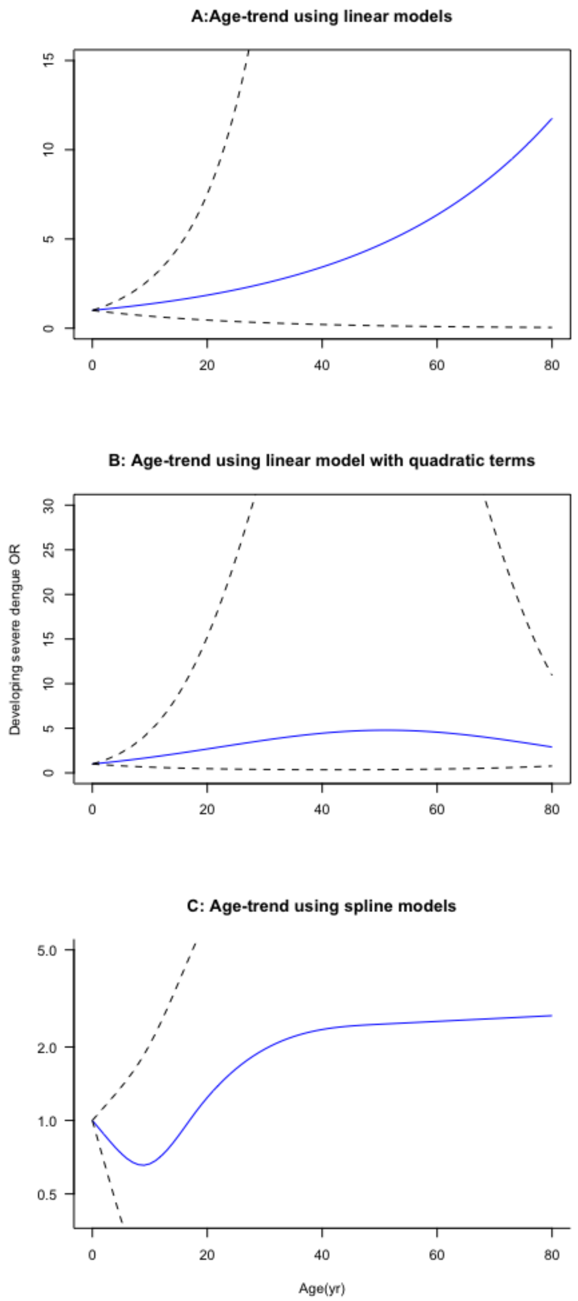

Supplement Figure 2: Association between odd ratios of severe progression (y-axis) and age (x-axis) from dose-response meta-analysis based on linear models (A), linear models with quadratic terms (B), and spline models with knots at age 2, 9, 18, and 50 (C). The blue solid line indicates the central estimate and the dotted line indicates the 95% CI.

Supplement Table 4: Model fitting parameters of linear and non-linear models estimating trends in the risk of developing severe manifestations with age in dose-response meta-analysis

Model	AIC	BIC
Linear model	65.8	67.6
Linear model with quadratic terms	83.1	87.3
Spline model with 4 knots at age 2, 9, 18, and 50	71.5	78.4

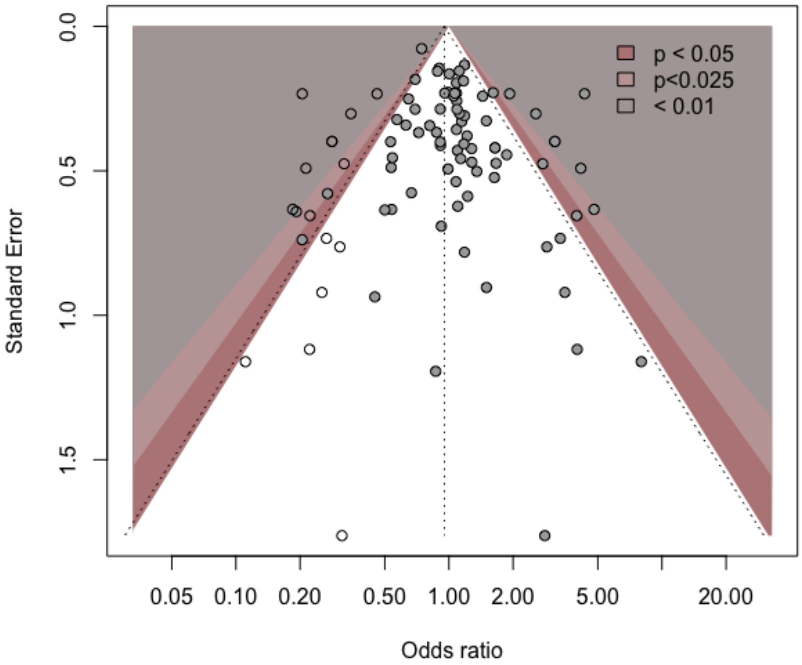

Supplement Figure 3: Funnel plot for association between sex and severe progression. The grey dots indicate association of each study and the clear dots indicate the imputed associations.

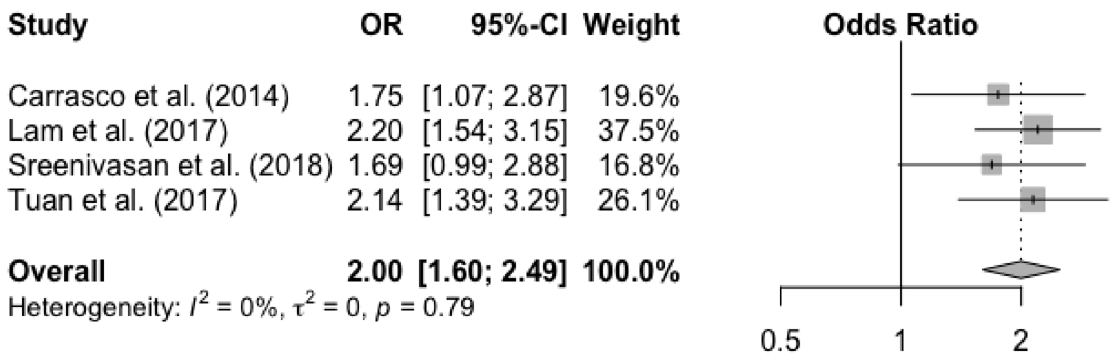

Supplement Figure 4: Forest plot of associations from multivariable models between vomiting and progression to severe disease. Four studies were included in the meta-analysis. The pooled OR was 2.00 (95% CI (1.60, 0.2.49)) and I^2 was 0%.

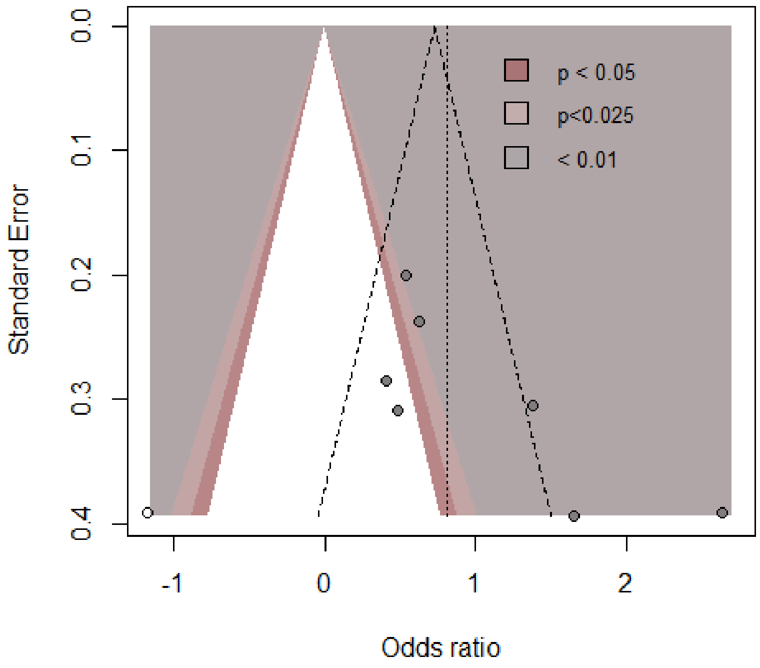

Supplement Figure 5: Funnel plot for association between AST and progression to severe disease. The grey dots indicate association of each study and the clear dots indicate the imputed associations. Dots on the coloured background indicate statistically significant ORs (red: p-value < 0.05, pink < 0.025 and brown p-value <0.01); dots on the white background indicate non-significant ORs (p-value ≥ 0.05).

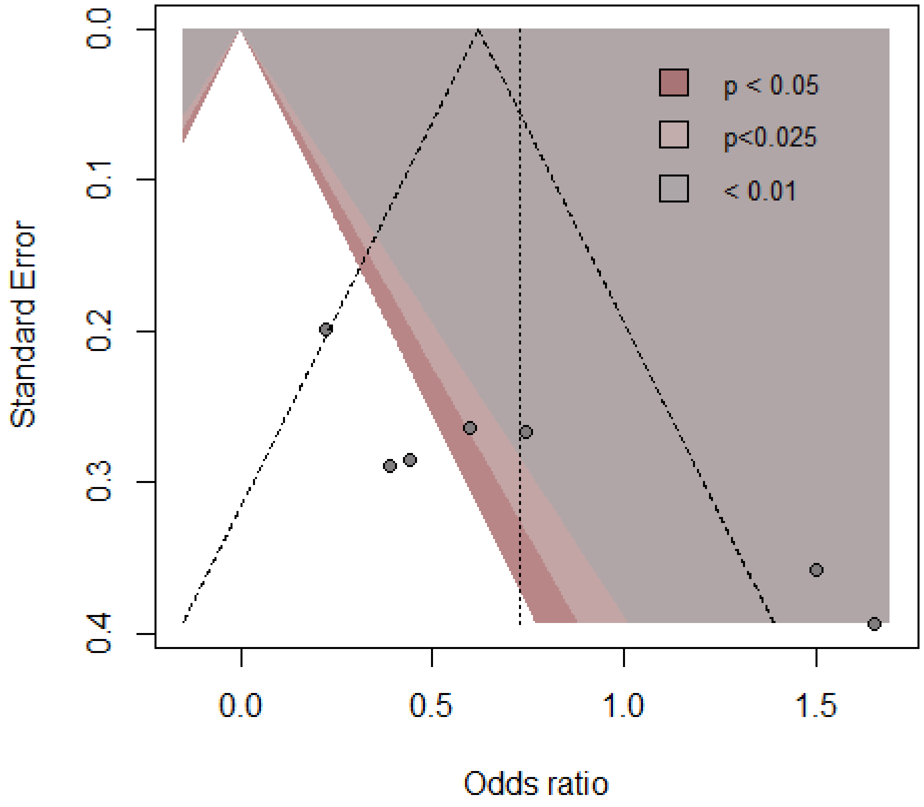

Supplement Figure 6: Funnel plot for association between ALT and progression to severe disease. The grey dots indicate association of each study. While dots on the colour background indicate statistically significant ORs (red: p-value < 0.05, pink < 0.025 and brown p-value <0.01), on white indicates non-significant.

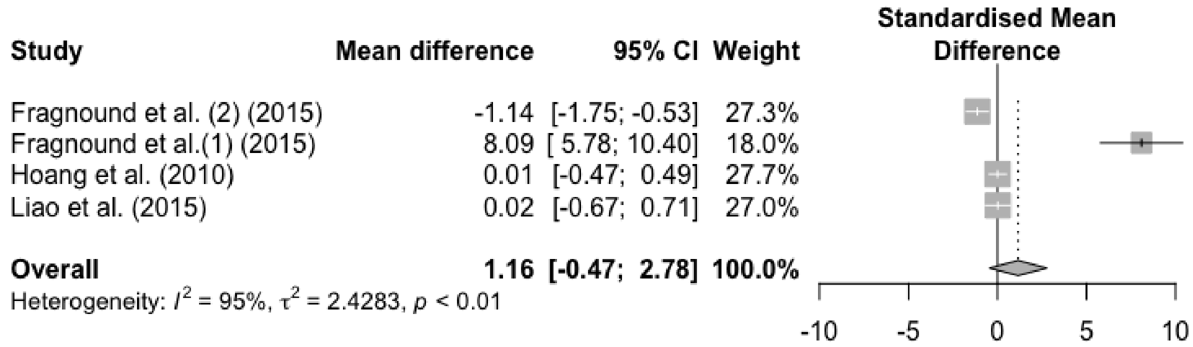

Supplement Figure 7: Forest plot of associations between viraemia and progression to severe disease. Four studies were included in the meta-analysis. The pooled OR was 1.16 (95% CI (-0.47 to 2.78)) and I^2 was 95%.

Supplement Table 7. Associated factors with progression to severe dengue illness reported in studies with children and adults

Associated factors reported in studies with children during the febrile phase of disease	Associated factors reported in studies with adults during the febrile phase of disease
1. Age<br><br>2. Sex<br><br>3. Nutritional status<br><br>4. Weight<br><br>5. Presence of vomiting<br><br>6. Presence of abdominal pain and tenderness<br><br>7. Presence of bleeding<br><br>8. Platelet count	1. Age<br><br>2. Sex<br><br>3. Mixed comorbidity<br><br>4. Diabetes mellitus<br><br>5. Chronic kidney disease<br><br>6. Cardiovascular disease<br><br>7. Platelet count<br><br>8. Haematocrit

9. Haematocrit<br><br>10. Aminotransferase levels<br><br>11. Immune status<br><br>12. Serotypes	9. Immune status<br><br>10. Serotype

Supplement Figure 8: Risk of bias in the included studies assessed by the Quality in Prognostic Studies tool

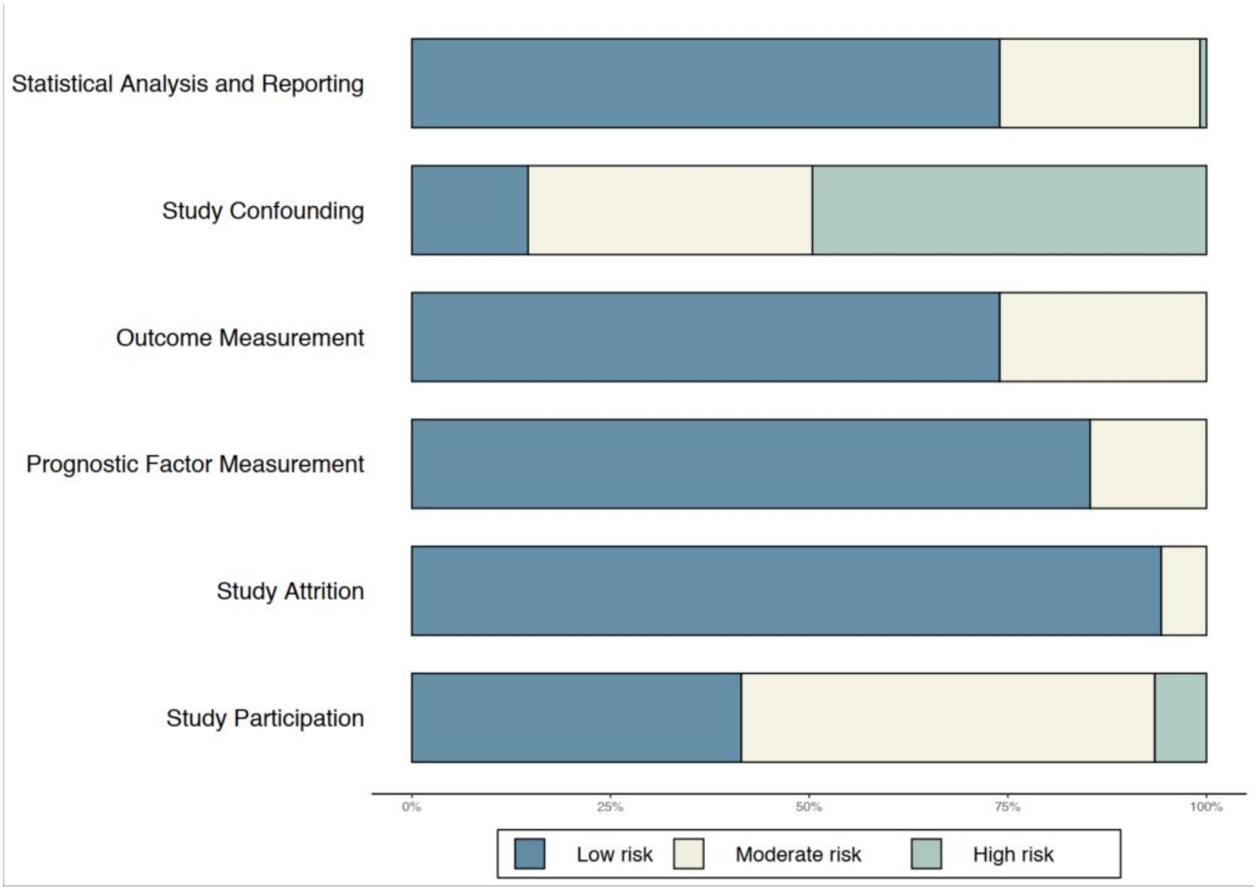

Supplement: Supplementary appendix [file mmc1.pdf]
